# Road traffic noise and breast cancer: DNA methylation in four core circadian genes

**DOI:** 10.1186/s13148-024-01774-z

**Published:** 2024-11-25

**Authors:** Jesse D. Thacher, Anastasiia Snigireva, Ulrike Maria Dauter, Mathilde N. Delaval, Anna Oudin, Kristoffer Mattisson, Mette Sørensen, Signe Borgquist, Maria Albin, Karin Broberg

**Affiliations:** 1 Division of Occupational and Environmental Medicine, Department of Laboratory Medicine, Lund University, Lund, Sweden; 2 Institute of Environmental Medicine, Karolinska Institutet, Stockholm, Sweden; 3 Joint Mass Spectrometry Centre (JMSC), Cooperation Group Comprehensive Molecular Analytics, Helmholtz Munich, Neuherberg, Germany; 4 Work, Environment and Cancer, Danish Cancer Institute, Copenhagen, Denmark; 5 Department of Natural Science and Environment, Roskilde University, Roskilde, Denmark; 6 Department of Oncology, Aarhus University Hospital, Aarhus University, Aarhus, Denmark; 7 Department of Clinical Sciences Lund, Oncology, Lund University and Skåne University Hospital, Lund, Sweden

**Keywords:** DNA methylation, Environmental noise, Road traffic noise, Traffic, Breast cancer, Sleep, Estrogen receptor

## Abstract

**Background:**

Transportation noise has been linked with breast cancer, but existing literature is conflicting. One proposed mechanism is that transportation noise disrupts sleep and the circadian rhythm. We investigated the relationships between road traffic noise, DNA methylation in circadian rhythm genes, and breast cancer. We selected 610 female participants (318 breast cancer cases and 292 controls) enrolled into the Malmö, Diet, and Cancer cohort. DNA methylation of CpGs (*N* = 29) in regulatory regions of circadian rhythm genes (*CRY1, BMAL1, CLOCK,* and *PER1*) was assessed by pyrosequencing of DNA from lymphocytes collected at enrollment. To assess associations between modeled 5-year mean residential road traffic noise and differentially methylated CpG positions, we used linear regression models adjusting for potential confounders, including sociodemographics, shiftwork, and air pollution. Linear mixed effects models were used to evaluate road traffic noise and differentially methylated regions. Unconditional logistic regression was used to investigate CpG methylation and breast cancer.

**Results:**

We found that higher mean road traffic noise was associated with lower DNA methylation of three *CRY1* CpGs (CpG1, CpG2, and CpG12) and three *BMAL1* CpGs (CpG2, CpG6, and CpG7). Road traffic noise was also associated with differential methylation of *CRY1* and *BMAL1* promoters. In *CRY1* CpG2 and CpG5 and in *CLOCK* CpG1, increasing levels of methylation tended to be associated with lower odds of breast cancer, with odds ratios (OR) of 0.88 (95% confidence interval (CI) 0.76–1.02), 0.84 (95% CI 0.74–0.96), and 0.80 (95% CI 0.68–0.94), respectively.

**Conclusions:**

In summary, our data suggest that DNA hypomethylation in *CRY1* and *BMAL1* could be part of a causal chain from road traffic noise to breast cancer. This is consistent with the hypothesis that disruption of the circadian rhythm, e.g., from road traffic noise exposure, increases the risk of breast cancer. Since no prior studies have explored this association, it is essential to replicate our results.

**Graphical abstract:**

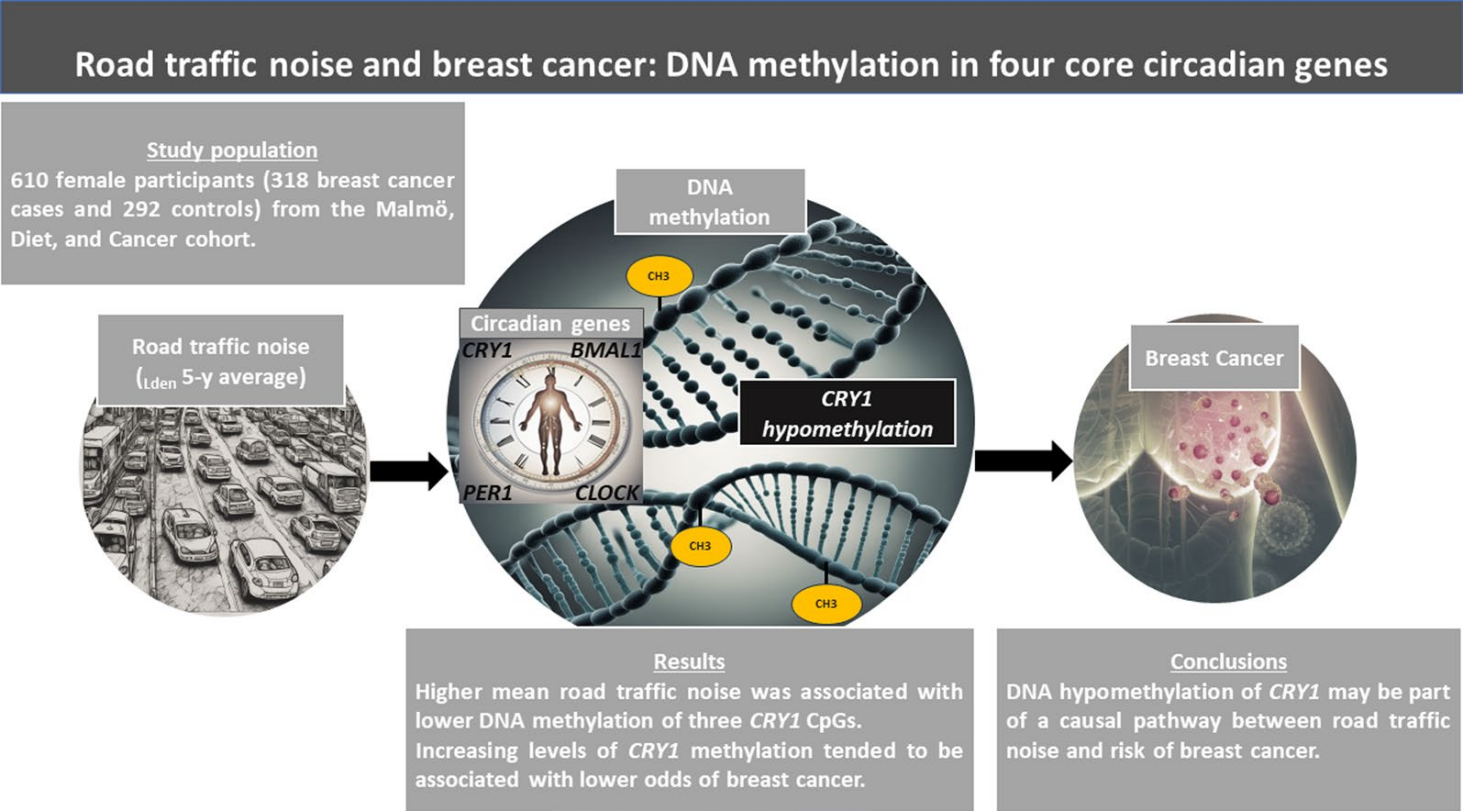

**Supplementary Information:**

The online version contains supplementary material available at 10.1186/s13148-024-01774-z.

## Background

Globally, breast cancer ranks as the most commonly detected malignancy among women [1]. Around five to ten percent of breast cancer cases are attributed to genetic factors and a variety of other risk factors have also been identified, including alcohol consumption, hormone replacement therapy, oral contraceptives, nulliparity, and mammographic density [2, 3]. Environmental exposures such as transportation noise [4–6] and traffic-related air pollution [7, 8] have been suggested to contribute to the etiology of breast cancer.

In Europe, transportation noise stands as the second most detrimental environmental risk factor contributing to ill health, surpassed only by air pollution [9]. More than 20% of the European Union's populace is exposed to transportation noise exceeding the recommended threshold of 55 dB (L_den_) [10], contributing to more than one million healthy life years lost per annum [11]. Transportation noise has been shown to increase the risk of cardiovascular and metabolic disease [12–19], and there is some evidence to indicate that transportation noise may be associated with breast cancer incidence [4–6]. Nevertheless, findings remain inconclusive, particularly with regard to estrogen receptor (ER) status. A recent study pooling eight Nordic cohorts reported an association for road traffic noise and breast cancer, with a 3% increased risk per 10-dB increase in 5-year mean noise, and with similar results among women with ER positive (ER +) and negative (ER-) breast cancer [4]. Additional cohort studies have reported inconsistent findings, with some studies reporting excess risk only in women with ER- breast cancer, whereas others reported associations mainly with ER + breast cancer [5, 6, 20].

The proposed mechanisms by which noise could impact breast cancer risk include sleep disturbances, both decreased sleep duration and poor quality, which can lead to the disruption of the biological rhythm [21–24]. The biological rhythm is regulated by the “master” circadian clock, which is generated and maintained in the suprachiasmatic nucleus (SCN) of the hypothalamus and regulates key physiological processes. Disturbance of the master circadian clock has been shown to be associated with cancer, and clock genes including the circadian locomotor output cycles kaput genes (CLOCK), basic helix–loop–helix ARNT-like genes (BMAL), period genes (PERs), and cryptochrome genes (CRYs) may influence critical functions in breast cancer etiology [25–29]. Furthermore, altered expression or function of clock regulatory factors has been implicated in certain types of cancer [28, 30] and specific genetic variations (polymorphisms) in CLOCK genes are also linked with breast cancer [26, 31, 32]. In a randomized crossover clinical study, one night of insomnolence was shown to change the epigenetic signature (i.e., gene regulatory as well as transcriptional) of core circadian clock genes in adipose tissue in humans [33]. In summary, traffic-induced sleep disturbance could lead to disrupted expression of CLOCK genes which in turn could lead to aberrant expression of genes in downstream pathways (e.g., hormone regulation and inflammatory response) ultimately contributing to breast cancer pathogenesis.

Overall, the molecular links between a potential effect of transportation noise on breast cancer risk are still not well understood, but altered gene regulation via DNA methylation of circadian genes may play a role. Therefore, we aimed to investigate the associations between long-term road traffic noise exposure, DNA methylation in four core circadian rhythm genes, and breast cancer, thus deepening knowledge on how traffic noise may increase breast cancer risk.

## Methods

### Study participants

The present study is performed in the Malmö Diet and Cancer Study (MDCS) which has been outlined elsewhere [34, 35]. In brief, 53,325 individuals were invited to take part in the study between 1991 and 1996. Criteria for inclusion were individuals living in Malmö, Sweden, and born between 1926 and 1945. In total, 30,446 subjects agreed to participate and comprised the study base.

At baseline, participants completed a questionnaire which included, but not limited to, questions on food consumption, lifestyle factors, reproductive history, occupation, and education level. Participants also underwent a health examination complemented with laboratory tests conducted by trained personnel. The health examination had a participation rate of 41%, of which 60% were females.

Based on the availability of DNA samples and financing for methylation analysis, a total of 610 female participants, consisting of 318 breast cancer cases (275 ER + , 43 ER −) and 292 controls, were available for the present study.

### Identification of cases

The Swedish National Cancer Registry contains information on all diagnosed malignant neoplasms in Sweden since 1958 [36]. By linking personal identification numbers to the cancer registry, we identified breast cancer cases. Incident cases were defined in accordance with the *International Classification of Diseases (ICD) eighth, ninth,* and *tenth revisions* as ICD8—174; ICD9—174; or ICD10—C50, respectively. Subsequently, cases were classified by estrogen receptor (ER) subtype, ER + and ER − , from the cancer register.

### Road traffic noise assessment

In the years 1990, 2000, and 2010, road traffic noise was estimated utilizing the Nordic Prediction Method implemented in SoundPLAN (version 8.0, SoundPLAN Nord ApS). For the present study, input variables included geocode, data on yearly mean diurnal traffic for all road links in Malmö municipality, vehicle distribution (heavy/light), signposted speed limits, diurnal distribution of traffic, and three-dimensional polygons for all buildings in Malmö. All road traffic sources within 1,000 m of receivers were incorporated. Traffic data were retrieved from a regional emission database [37]. The screening effects from buildings were included and ground softness considered. Terrain was not included, as Malmö is relatively flat. The parameter setting in the models was set to allow for two reflections and receivers placed at a two-meter height. For intermediate years, using the three models from 1990, 2000, and 2010, exposure assignment was made based on residential address for the year closest in time or year of major infrastructure changes. The equivalent continuous A-weighted sound pressure level (L_Aeq_) at the most exposed facade of the residence was calculated and expressed as L_den_, which is the mean for day (L_day_; 0700–1900 h), evening (L_evening_; 1900–2200 h), and night (L_night_; 2200–0700 h). Five- and ten-dB penalties were added to evening and night, respectively. Road traffic noise levels below 35 dB were assumed to be the lower limit of ambient noise and assigned a value of 35 dB. In the present study, we investigated mean residential road traffic noise exposure in 5-year time periods preceding baseline.

### DNA methylation

DNA from peripheral blood lymphocytes was extracted utilizing the E.Z.N.A. Blood kit (D3392-02, Omega Bio-Tek, USA). Sample preparation included the following steps: bisulfite treatment, PCR amplification, and pyrosequencing. Bisulfite treatment was completed with the EZ-96 DNA Methylation Gold kit (D5008, Zymo Research, USA). The PyroMark PCR system (Qiagen, Hilden, Germany) was used to generate specific PCR products. Bisulfite-treated template DNA (20 ng) was added to 12.5 µL of PyroMark PCR Master Mix (Qiagen), 2.5 µL of the forward and reverse primers set (140 nM), and water to set up a 25 µL PCR. The lists of primer sequences as well as PCR conditions are presented in the supplemental material (Supplementary file 1: Table S1). All PCR protocols contained 45 cycles.

The entire pyrosequencing analysis was completed utilizing the Pyromark Gold Q96 kit (Qiagen). Twenty µL of PCR product was incubated first with streptavidin sepharose high-performance beads (Cytiva, Uppsala, Sweden); subsequently, the biotin-labeled single-stranded DNA was purified, rinsed with 70% EtOH, denatured with 0.2 M NaOH, and rinsed again with wash buffer (Qiagen). Following elution, the DNA was temporarily incubated in an annealing mixture including the sequencing primer (0.4 µM); the plates were then heated to a maximum of 80 degrees Celsius for two minutes. The pyrosequencing assay was run in duplicates. Each pyrosequencing run included bisulfite-treated methylated and unmethylated DNA controls and negative controls. The CpG sites in *CRY1, BMAL1, CLOCK*, and *PER1* were situated in the proximal promoter regions (Supplementary file 1: Fig. S1) and were selected for analysis based on putative transcription factor binding information [38].

Degree of methylation at each CpG position was operationalized as the percentage of methylated cytosines, defined as the frequency of methylated cytosines divided by the total number of methylated and unmethylated cytosines. The percentage of DNA methylation was subsequently converted to M − values for each respective CpG site using the following formula, M_i_ = log_2_ $\left(\frac{p{\text{i}}}{1-pi}\right)$ [39].

### Covariates

Covariate selection was conducted a priori guided by biological plausibility, current literature, and availability. Confounders were assessed at baseline and included age, education level (low, medium, high), parity (nulliparous/parous), physical activity (low, medium, high), civil status (single/divorced/widow(er), married/cohabiting), occupational status (employed, unemployed, retired), smoking status (current, former, never), alcohol consumption (grams/day), and inconvenient working hours or shiftwork (yes, no). Body mass index (BMI) was also included and was measured as kilograms/meters^2^.

Traffic-related air pollution (NO_x_ and PM_2.5_) was modeled utilizing EnviMan (Opsis AB, Sweden) implementing a Gaussian dispersion model (AERMOD) and is described in detail elsewhere [37]. In short, the 18 × 18-km modeling area covered the city of Malmö and the surroundings. Emission data were gathered for the years 1992, 2000, and 2011 from preexisting regional as well as local databases maintained by the municipality. Annual average concentrations were stored as grids at a resolution of 50 × 50 m. Linear interpolation was applied to calculate intermediate years with adjustment for fluctuations in local meteorological conditions. Exposure data were combined with geocoded addresses to assign each participant annual residential exposure [37, 40].

### Statistical analysis

The correlations between the DNA methylation levels at each CpG site for each respective gene were assessed by using a Spearman correlation matrix. In crude and adjusted models, unconditional logistic regression models were used to assess associations between M − values of DNA methylation and breast cancer. Additionally, we used linear regression analyses to examine associations between a per 10-dB increase in 5-year time-weighted average road traffic noise at baseline and methylation for each CpG. Analyses were stratified by breast cancer cases and controls as well as by ER + and ER − breast cancer status. We conducted linear mixed effects models to assess the associations between road traffic noise and differently methylated regions (DMRs) of each selected gene. The mixed effects models included CpG site as random factor and road traffic noise as a fixed factor.

Categories of methylation (no methylation, below/above median methylation) and risk of breast cancer were evaluated post hoc in logistic regression models in genes and CpGs that were associated with noise in all, among the cases, or non-cases.

Two models were calculated—one model adjusting for age and a second fully adjusted model including age, parity, physical activity, education level, civil status, occupational status, smoking status, and alcohol consumption.

As a sensitivity analysis, we assessed the influence of further adjustment for additional possible confounders or mediators, in particular BMI, inconvenient working hours or shiftwork, PM_2.5_, and NO_x_. In addition, as traffic noise impacts sleep, we examined the association between self-reported sleep (difficulties falling asleep and staying asleep) and DNA methylation as well as breast cancer risk.

All analyses were conducted in SAS, version 9.4 (SAS Institute Inc., Cary, NC).

## Results

The distribution of covariates at baseline among all participants as well as stratified by cases and controls is presented in Table 1. Overall, breast cancer cases were less likely to be parous, menopausal, have low education, single, unemployed, physically active, work inconvenient hours, and active smokers compared to controls. Spearman correlations among CpG sites within each respective gene ranged up to a maximum of 0.74 (*CRY1* CpG7 and CpG10) and 0.46 among CpG sites across genes (*BMAL1* CpG6 and *CLOCK* CpG3) (Supplementary file 1: Fig. S2). Descriptive statistics of methylation of CpG sites in *CRY1, BMAL1, CLOCK,* and *PER1* among all participants, cases, and controls are presented in Tables S2 (Supplementary file 1), respectively. In general, DNA methylation across the four genes was minimal (median < 7%). No apparent differences in mean and median levels of methylation were observed across all participants, cases and controls (Supplementary file 1: Table S2).

**Table 1:** Baseline sociodemographic characteristics of the study population

Baseline characteristics	Total (N = 610)	Non-cases (n = 292)	All breast cancer cases (n = 318)	ER + breast cancer (n = 275)	ER– breast cancer (n = 43)
Age, years (mean ± SD)	56.3 ± 7.3	56.6 ± 7.3	55.9 ± 7.1	55.8 ± 7.1	56.9 ± 7.1
5-year mean road traffic noise at baseline, median (5–95%)	54.2 (40.4–67.4)	54.7 (41.0–67.5)	54.0 (40.0–66.9)	54.2 (40.3–66.9)	54.1 (38.6–68.1)
Parity, %					
Nulliparous	13.9	12.7	15.1	14.9	16.3
Parous	86.1	87.3	84.9	85.1	83.7
Age at first birth	24.8 ± 4.6	24.6 ± 4.4	25.0 ± 4.7	24.9 ± 4.5	25.6 ± 6.2
Menopause, %					
Still menstruating	30.2	28.8	31.5	32.4	25.6
Menopausal	67.2	68.8	65.7	65.1	69.8
Unknown	2.6	2.4	2.8	2.5	4.7
Educational level, %					
Low	67.2	69.2	65.4	65.1	67.4
Medium	15.9	16.4	15.4	15.3	16.3
High	16.9	14.4	19.2	19.6	16.3
Civil status, %					
Single/divorced/ widow(er)	37.0	40.7	33.6	32.4	41.9
Married/cohabiting	63.0	59.3	66.4	67.6	58.1
Occupational status (%)					
Gainfully employed	68.9	66.8	70.8	71.3	67.4
Unemployed	7.5	8.2	6.9	6.9	7.0
Retired	23.6	25.0	22.3	21.8	25.6
Physical activity, %					
Low	50.8	48.3	53.1	52.0	65.1
Medium	21.9	24.0	20.1	19.6	23.3
High	26.3	26.7	26.8	28.4	11.6
Inconvenient working hours/shiftwork, %					
Yes	23.8	26.7	21.1	22.2	14.3
No	75.1	71.9	78.0	77.8	85.7
BMI (kg/m^2^), mean ± SD	25.3 ± 3.9	25.1 ± 3.9	25.4 ± 4.0	25.5 ± 4.0	24.7 ± 3.8
Waist circumference (cm), mean ± SD	77.4 ± 10.2	77.1 ± 10.0	77.6 ± 10.4	77.9 ± 10.6	76.3 ± 8.8
Smoking, %					
Current	25.1	31.2	19.5	20.7	11.6
Former	29.5	23.6	34.9	34.9	34.9
Never	45.4	45.2	45.6	44.4	53.5
Smoking intensity (g/day)^b^, mean ± SD	12.1 ± 6.7	11.4 ± 5.9	13.1 ± 7.6	12.9 ± 7.5	14.8 ± 9.6
Alcohol intake (g/day)^b^, mean ± SD	10.7 ± 9.6	10.8 ± 9.6	10.6 ± 9.7	10.8 ± 9.9	9.1 ± 8.2
PM_2.5_ (µg/m^3^)^c^, mean ± SD	9.8 ± 2.4	9.8 ± 2.4	9.7 ± 2.4	9.8 ± 2.3	9.5 ± 3.3
NO_x_ (µg/m^3^)^c^, mean ± SD	36.2 ± 13.6	36.6 ± 13.3	35.8 ± 13.9	35.4 ± 13.1	38.8 ± 18.2

*SD* standard deviation

^a^Among women with ≥ 1 birth

^b^Among exposed

^c^At baseline

Overall, a 10 dB increase in 5-year mean road traffic noise was associated with lower DNA methylation in *CRY1* CpG2 and three *BMAL1* CpGs (CpG2, CpG6, and CpG7) (Table 2). No consistent associations were present across breast cancer cases and non-cases in relation to road traffic noise and DNA methylation. However, among breast cancer cases, road traffic noise tended to be more strongly associated with lower methylation in *CRY1* CpG1, CpG2, CpG4, CpG6, and CpG12, with betas ranging from -0.30 to -0.19 (Table 2).

**Table 2:** Associations between road traffic noise (linear, per 10-dB increase in 5-y mean exposure at baseline) and DNA methylation

Gene/CpG	Road traffic noise and methylation		
	All^a^	Non-cases^a^ n = 292	Cases^a^ n = 318
	Beta (SE), *p-value*	Beta (SE), *p-value*	Beta (SE), *p-value*
*CRY1* CpG1	−0.13 (0.07), 0.06	−0.05 (0.10), 0.59	−0.22 (0.10), 0.03
*CRY1* CpG2	−0.17 (0.07), 0.01	−0.05 (0.09), 0.59	−0.30 (0.10), 0.002
*CRY1* CpG3	−0.05 (0.07), 0.45	0.06 (0.10), 0.55	−0.17 (0.10), 0.11
*CRY1* CpG4	−0.08 (0.07), 0.25	0.02 (0.10), 0.84	−0.21 (0.10), 0.04
*CRY1* CpG5	−0.03 (0.08), 0.71	0.01 (0.11), 0.95	−0.05 (0.11), 0.68
*CRY1* CpG6	−0.09 (0.05), 0.08	0.01 (0.07), 0.95	−0.19 (0.07), 0.01
*CRY1* CpG7	−0.02 (0.06), 0.70	0.07 (0.09), 0.43	−0.14 (0.10), 0.16
*CRY1* CpG8	−0.07 (0.07), 0.27	0.02 (0.09), 0.83	−0.16 (0.10), 0.09
*CRY1* CpG9	−0.12 (0.07), 0.12	−0.03 (0.10), 0.74	−0.21 (0.11), 0.06
*CRY1* CpG10	−0.10 (0.08), 0.24	−0.01 (0.11), 0.93	−0.20 (0.12), 0.10
*CRY1* CpG11	−0.04 (0.06), 0.53	0.07 (0.08), 0.36	−0.15 (0.09), 0.09
*CRY1* CpG12	−0.14 (0.07), 0.05	−0.11 (0.10), 0.27	−0.20 (0.10), 0.05
*BMAL1* CpG1	−0.02 (0.06), 0.68	0.03 (0.08), 0.68	−0.09 (0.09), 0.32
*BMAL1* CpG2	−0.13 (0.05), 0.01	−0.13 (0.07), 0.07	−0.12 (0.07), 0.09
*BMAL1* CpG3	−0.08 (0.06), 0.18	−0.13 (0.08), 0.09	−0.04 (0.09), 0.61
*BMAL1* CpG4	0.01 (0.05), 0.95	−0.01 (0.07), 0.97	0.02 (0.07), 0.83
*BMAL1* CpG5	−0.04 (0.05), 0.38	−0.04 (0.07), 0.53	−0.02 (0.07), 0.73
*BMAL1* CpG6	−0.12 (0.05), 0.01	−0.15 (0.08), 0.07	−0.11 (0.07), 0.12
*BMAL1* CpG7	−0.13 (0.06), 0.04	−0.20 (0.09), 0.02	−0.04 (0.08), 0.61
*CLOCK* CpG1	−0.04 (0.06), 0.48	−0.11 (0.08), 0.15	0.03 (0.08), 0.70
*CLOCK* CpG2	−0.02 (0.06), 0.68	0.04 (0.09), 0.62	−0.12 (0.08), 0.17
*CLOCK* CpG3	−0.04 (0.06), 0.51	−0.08 (0.09), 0.32	0.02 (0.08), 0.82
*CLOCK* CpG4	−0.03 (0.06); 0.57	−0.02 (0.09), 0.85	−0.08 (0.09), 0.35
*CLOCK* CpG5	−0.07 (0.06), 0.18	−0.04 (0.08), 0.59	−0.09 (0.08), 0.25
*PER1* CpG1	−0.03 (0.06), 0.61	−0.01 (0.08), 0.97	−0.07 (0.08), 0.38
*PER1* CpG2	−0.02 (0.06), 0.78	0.02 (0.08), 0.79	−0.04 (0.09), 0.63
*PER1* CpG3	0.02 (0.06), 0.68	0.11 (0.08), 0.14	−0.08 (0.09), 0.39
*PER1* CpG4	−0.05 (0.07), 0.46	0.04 (0.09), 0.66	−0.16 (0.10), 0.10
*PER1* CpG5	−0.01 (0.02), 0.52	−0.01 (0.03), 0.68	−0.01 (0.03), 0.77

*CI* confidence interval; *SE* standard error

^a^Adjusted for age, parity, physical activity, education level, civil status, occupational status, smoking status, and alcohol consumption

We observed an inverse association between road traffic noise and differentially methylated regions of *CRY1* and *BMAL1* among cases and non-cases in mixed-effects models, Beta = −0.07 (95% CI: −0.11 to -0.03) and Beta = −0.06 (95% CI: −0.09 to −0.02), respectively (Table 3). For *CRY1* the association appeared to be strongest among breast cancer cases, Beta = −0.18 (95% CI: −0.24 to −0.12). No associations were apparent between road traffic noise and differentially methylated regions of *CLOCK* or *PER1,* Beta = −0.03 (95% CI: −0.08 to 0.02) and Beta = -0.01 (95% CI −0.06 to 0.04), respectively (Table 3).

**Table 3:** Associations between road traffic noise (linear, per 10-dB increase in 5-y mean exposure at baseline) and differently methylated regions of *CRY1*, *BMAL1*, *CLOCK*, and *PER1*

Gene	All		Non-cases	Cases
	Crude Models^a^	Adjusted Models^b^	Adjusted Models^b^	Adjusted Models^b^
	Beta (95% CI), *p-value*	Beta (95% CI), *p-value*	Beta (95% CI), *p-value*	Beta (95% CI), *p-value*
*CRY1*	−0.05 (−0.09 to −0.01), 0.01	−0.07 (−0.11 to−0.03), < 0.001	0.02 (−0.04 to 0.07), 0.55	−0.18 (−0.24 to −0.12), < 0.0001
*BMAL1*	−0.05 (−0.09 to −0.01), 0.01	−0.06 (−0.09 to −0.02), 0.01	−0.07 (−0.13 to −0.02), 0.01	−0.04 (−0.10 to 0.02), 0.16
*CLOCK*	−0.01 (−0.06 to 0.04), 0.59	−0.03 (−0.08 to 0.02), 0.27	−0.02 (−0.10 to 0.05), 0.53	−0.04 (−0.11 to 0.03), 0.30
*PER1*	−0.02 (−0.06 to 0.03), 0.48	−0.01 (−0.06 to 0.04), 0.79	0.03 (−0.04 to 0.10), 0.36	−0.06 (−0.13 to 0.02), 0.13

*CI* confidence interval

^a^Adjusted for age

^b^Adjusted for age, parity, physical activity, education level, civil status, occupational status, smoking status, and alcohol consumption

**Fig. 1:**
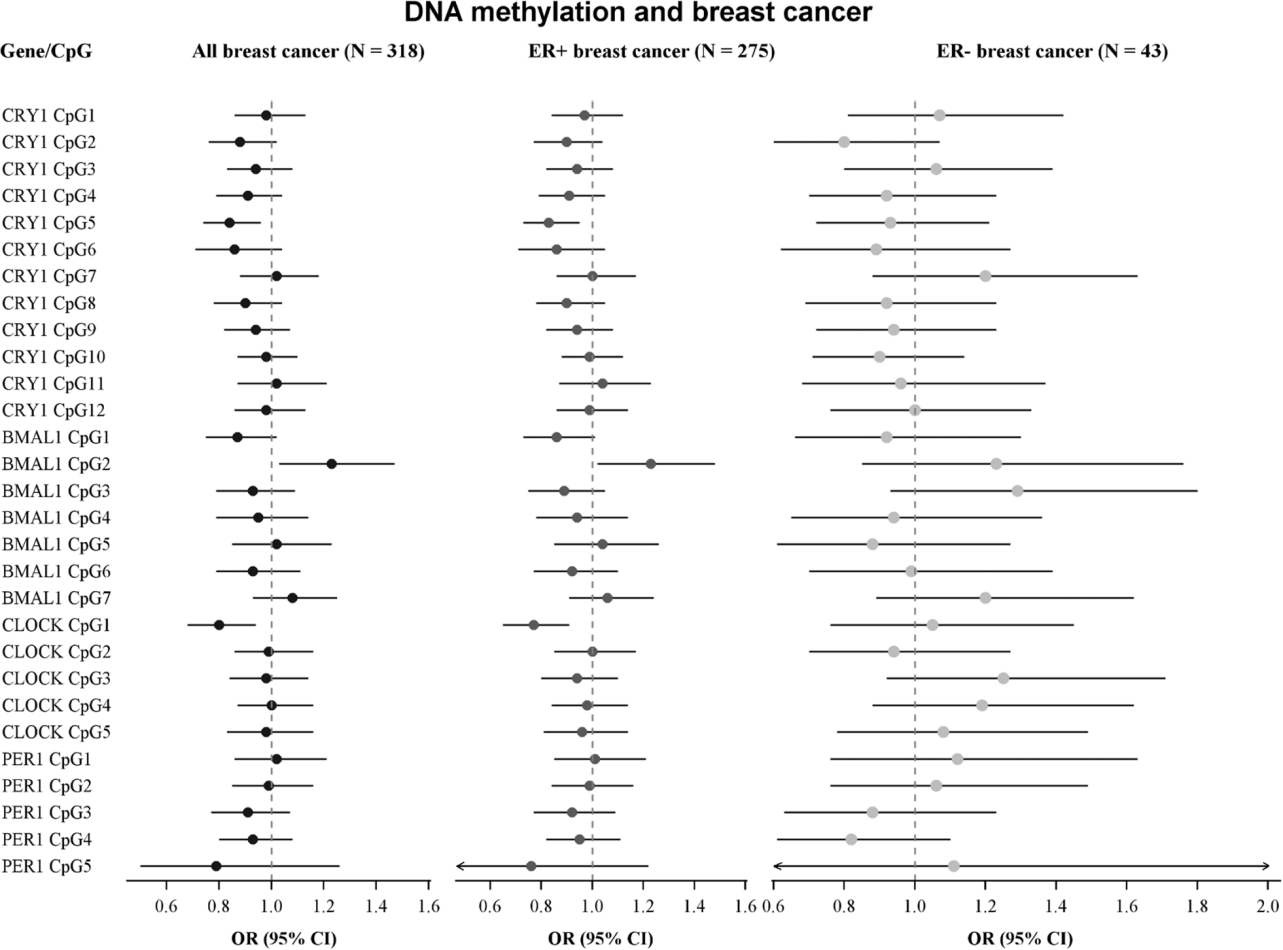
Associations between DNA methylation and breast cancer. Adjusted for age, parity, physical activity, education level, civil status, occupational status, smoking status, and alcohol consumption

Overall, no consistent patterns between DNA methylation and breast cancer were observed. Nevertheless, in *CRY1* CpG2 and CpG5 and in *CLOCK* CpG1 increasing levels of methylation tended to be associated with lower odds of breast cancer (OR 0.88; 95% CI 0.76–1.02, OR 0.84; 95% CI 0.74–0.96, and OR 0.80; 95% CI: 0.68–0.94, respectively) (Fig. 1). Contrastingly, DNA methylation in *BMAL1* CpG2 was associated with breast cancer (OR 1.23; 95% CI 1.03–1.47).

**Fig. 2:**
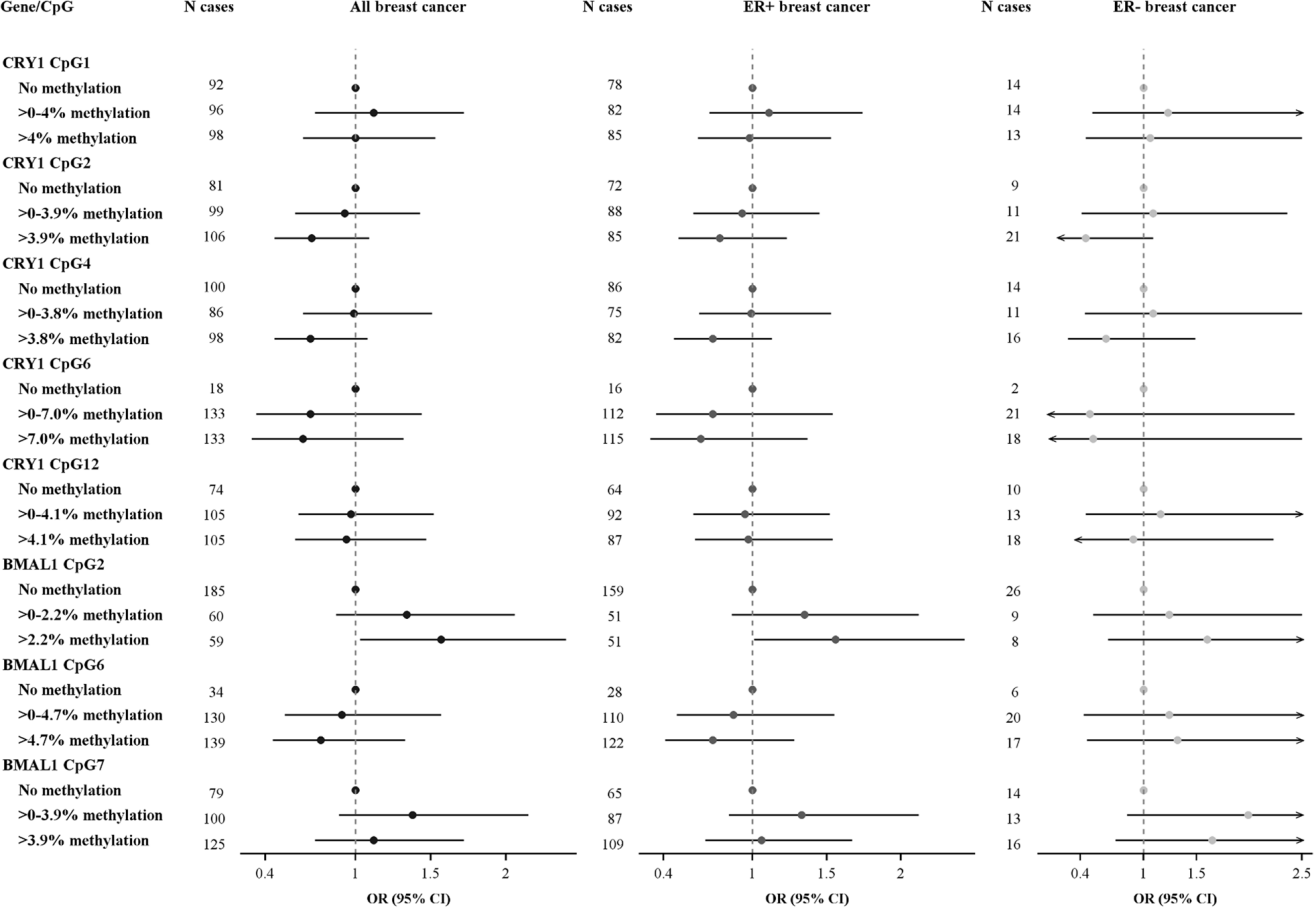
Associations between levels of *CRY1* and *BMAL1* methylation and breast cancer in adjusted logistic regression models. Adjusted for age, parity, physical activity, education level, civil status, occupational status, smoking status, and alcohol consumption. Categorization based on above/below median methylation values among those with any methylation

In post hoc analyses, we evaluated the effect of categorized methylation in *CRY1* (CpG1, CpG2, CpG4, CpG6, CpG12) and *BMAL1* (CpG2, CpG6, CpG7) and breast cancer. Overall, no clear associations between DNA methylation and breast cancer were observed (Fig. 2).

We found no marked differences in the association between DNA methylation and risk of ER + compared to ER − breast cancer (Fig. 1).

Associations between road traffic noise and DNA methylation as well as DNA methylation and breast cancer did not differ substantially between crude and adjusted models (Supplementary file 1: Tables S3 and S4, respectively).

In sensitivity analyses, additional adjustment for PM_2.5_, NO_x_, inconvenient working hours, or BMI had little effect on effect estimates (Supplementary file 1: Tables S5–S8, respectively). Additionally, among those with available information (*n* = 413), no clear patterns between self-reported problems falling sleep or problems staying asleep and DNA methylation were noted (Supplementary file 1: Tables S9 and S10, respectively). Similarly, falling or staying asleep was not consistently associated with breast cancer risk (Supplementary file 1: Table S11).

## Discussion

This is the first epidemiological study evaluating the associations between long-term road traffic noise, DNA methylation, and breast cancer. Road traffic noise appeared to be inversely associated with regional changes of *CRY1* and *BMAL1*, and specifically hypomethylation in *CRY1* CpG1, CpG2, and CpG12 as well as *BMAL1* CpG2, CpG6, and CpG7. In addition, some indication of DNA methylation being inversely associated with breast cancer risk suggests that DNA hypomethylation in certain circadian genes may be part of a causal chain from road traffic noise to breast cancer pathogenesis.

Epidemiological studies examining the association between transportation noise and epigenetic changes are limited. In a Swiss EWAS study, traffic noise demonstrated primarily decreased methylation at specific DMRs, which is somewhat in line with the present study where we found road traffic noise to be associated with hypomethylation in multiple *CRY1* and *BMAL1* CpGs [41]. Additionally, in the brains of rats, long-term nocturnal noise was associated with aberrant methylation, in particularly hypomethylation of the melanocortin 2 receptor (*Mc2r*) gene in the hippocampus [42]. Further evidence from murine models demonstrated that murine cochlea and inferior colliculus contain circadian machinery and that noise exposure differentially impacted the expression of core clock genes in the auditory periphery and inferior colliculus [43, 44]. Both *CRY1* and *BMAL1* are core components of the circadian clock, along with other period genes, and orchestrate the circadian rhythm through the complex interplay involving positive and negative feedback loops, self-expression regulation, as well as additional axillary regulatory processes [45]. In short, BMAL and CLOCK transcription factors form the heterodimer that promotes the expression of CLOCK and CLOCK-regulated genes. Conversely, PER and CRY constitute the inhibitory complex which impedes the CLOCK-BMAL protein complex [29, 38].

The consequences of our results need to be elucidated since DNA hypermethylation is frequently linked with transcriptional gene repression, while hypomethylation is often linked with a chromatin arrangement that supports transcription [46]. It is unclear what the methylation changes observed in the present study are predicted to result in, but overexpression and aberrant expression of certain circadian genes have been found in cancer tissue, including breast cancer [47]. Thus, it is conceivable that long-term road traffic noise could lead to altered gene transcription and expression, hallmarks in multiple cancers, including breast cancer. Therefore, further research should evaluate other cancer hallmarks (e.g., inflammation, apoptosis, or DNA repair) in relation to traffic noise and breast cancer risk.

We observed some indication that methylation of multiple CpGs in *CRY1* and *CLOCK* was inversely associated with breast cancer. *CRY1*’s role in breast cancer development is not fully understood; however, *CRY* is involved in regulation of DNA replication, DNA damage, and cell cycle [48, 49]. *CRY1*, in particular, is also a known regulator of cell proliferation and DNA repair [50] and has been shown to inhibit nuclear receptors involved in certain cancers [51]. Two studies demonstrated a link between hypermethylation of the *CLOCK* gene with lower breast cancer risk which is congruent with our findings [52, 53]. Increased methylation might result in reduced gene expression, consequently weakening *CLOCK* proliferation. Furthermore, *CLOCK* and *CRY1* might possess tumorigenic characteristics, and this is substantiated by whole-genome expression microarray studies that found expression of multiple cancer-related transcripts to be modified after *CLOCK* gene knockdown. More specifically, after silencing the *CLOCK* gene, the genes primarily involved in breast cancer progression included *CCL5* [54], *SP100* [55], and *BDKRB2* [56].

In summary, the aforementioned factors reveal a potential pathway from road traffic noise to dysregulation of the circadian clock and breast carcinogenesis. Nevertheless, the mechanism from noise to circadian rhythm disruption and the development of breast cancer remain to be fully elucidated.

Road traffic noise and traffic-related air pollution are correlated since they share some of the same emission sources, and air pollution has also been linked to both DNA methylation and breast cancer risk [57, 58]. Therefore, it is crucial for research on noise exposure to take into consideration air pollution, and conversely, for studies on air pollution to consider traffic noise exposure. In the present study, estimates for road traffic noise and methylation, as well as for DNA methylation and breast cancer, were not impacted to any large extent when adjusting for PM_2.5_ or NO_x_.

We opted to not adjust for multiple comparisons since the CpGs are intercorrelated, particularly for *CRY1*, and therefore would result in overadjustment.

A key strength of the present study is that it is based on a well-characterized cohort which includes data on many potential confounders, namely air pollution and inconvenient working hours. Another strength is that we focused on DNA methylation in specific genes related to both sleep disturbance and breast cancer. Lastly, we utilized pyrosequencing (DNA sequencing) and it is considered the benchmark for analyzing DNA methylation.

Although our findings suggest that long-term road traffic noise potentially results in epigenetic changes in circadian genes, the molecular pathomechanisms underlying this phenomenon remain obscure. An important limitation is that our findings are based on a limited sample size and further studies to corroborate our findings are recommended. Furthermore, we assessed methylation levels of 29 CpGs, and it is possible that this number was insufficient to establish a link between noise and cancer via circadian disruption. Although we selected multiple CpGs in the promoter regions harbored within CpG islands, it should be noted that transcriptional regulation is a complex process, and regulation can impact various locations throughout gene bodies. Therefore, the link between traffic noise, DNA methylation, and breast cancer potentially involves other parts of the CpG islands outside the currently selected regions. Nonetheless, the selected CpGs were in the promoter region of the genes, which regulate gene expression, and aberrant methylation of these CpGs may have large functional impact. Another limitation of our study is that we measured DNA methylation in lymphocytes and not the brain or breast. However, circadian clocks are present in most cells throughout the body. We cannot rule out residual confounding from unaccounted risk factors. For example, we lack information on artificial light at night, which could potentially bias our findings, as light at night is associated with disruption of the circadian rhythm and has been purported as a possible mechanism of cancer etiology [59]. Lastly, given the moderate amount of missing data on self-reported sleep difficulties as well as the inability to assess other sleep qualities (e.g., feeling rested) in our sample, these findings should be interpreted with caution.

## Conclusions

In conclusion, our findings suggested that DNA hypomethylation in certain CpG sites of *CRY1* may be part of a causal pathway between road traffic noise and risk of breast cancer. This is consistent with the hypothesis that disruption of the circadian rhythm, e.g., through road traffic noise exposure, increases the risk of breast cancer. Our findings, although exploratory, contribute to the very limited evidence base regarding traffic noise and gene alterations and demonstrate some evidence of breast cancer-relevant epigenetic effects of transportation noise.

## Supplementary Information

Additional file 1.

## Data Availability

The datasets generated and/or analyzed during the current study are available from the Lund University Medical Faculty—Malmo Diet and Cancer Cohort, but restrictions apply to the availability of these data.

## Abbreviations

BMAL: Basic helix–loop–helix ARNT-like genes
BMI: Body mass index
CLOCK: Circadian locomotor output cycles kaput genes
CRYs: Cryptochrome genes
dB: Decibel
ER: Estrogen receptor
ICD: International Classification of Diseases
L_Aeq_: Energy-equivalent average A-weighted sound pressure level
L_den_: The weighted day–evening–nighttime average noise indicator over 24-h period
MDCS: Malmö Diet and Cancer Study
NO_x_: Oxides of nitrogen
PERs: Period genes
PM: Particulate matter
SCN: Suprachiasmatic nucleus
