# Supplementary material for: Road traffic noise and breast cancer: DNA methylation in four core circadian genes
Source: Clin Epigenetics. 2024 Nov 25;16:168. doi: 10.1186/s13148-024-01774-z (PMC11590349; doi:10.1186/s13148-024-01774-z)
Supplement: Supplementary file 1 — Additional file 1. [file 13148_2024_1774_MOESM1_ESM.docx]

**SUPPLEMENTAL TABLES AND FIGURES**

Road traffic noise and breast cancer: DNA methylation in four core circadian genes

| Table S1. Primer sequences and polymerase chain reaction conditions. | | | | | | |
| --- | --- | --- | --- | --- | --- | --- |
| Gene | **Gene location** | **Number of CpG sites** | **Forward primer** | **Reverse primer** | **Pyrosequencing primer** | **PCR conditions** |
| CRY1 | 12q23.3 | 12 | GGTTTTTTGTGAGGGAAGGTTTAGT | ACCCCCCCCCCTTACCCTCTA | GGGATGAGGGGAGTT | 94°C for 30s, 58°C for 30s, 72°C for 30s |
| ARNTL/BMAL1 | 11p15.3 | 7 | AGTAGGAGGGAGAGAGGGAGTTA | CCCCTACCCTCTCCCTTTC | ATTTAGAGAAGAGGGATAT | 94°C for 30s, 59.4°C for 30s, 72°C for 30s |
| CLOCK | 4q12 | 5 | TTTTTGGGTAGAAATTTGGTTTTGTAG | TCTAACCTCTAAATCCCTCATCC | GTTTGGTAGGGTGGAG | 94°C for 30s, 56°C for 30s, 72°C for 30s |
| PER1 | 17p13.1 | 5 | GCGTGGGGGGCGTGGCAGCCCGGCCCGTGGGCGGGT | GYGTGGGGGGYGTGGTAGTTYGGTTYGTGGGYGGGT | TGTCGTGGTCGATGTCATGTCGTCGTAGTCG | 94°C for 30s, 56°C for 30s, 72°C for 30s |

**Figure S1.** Visualization of gene and CpG locations for *CRY1*, *BMAL1*, *CLOCK*, and *PER1* (figure from <https://genome.ucsc.edu/>). Red arrows indicate the approximate regions of the CpGs.


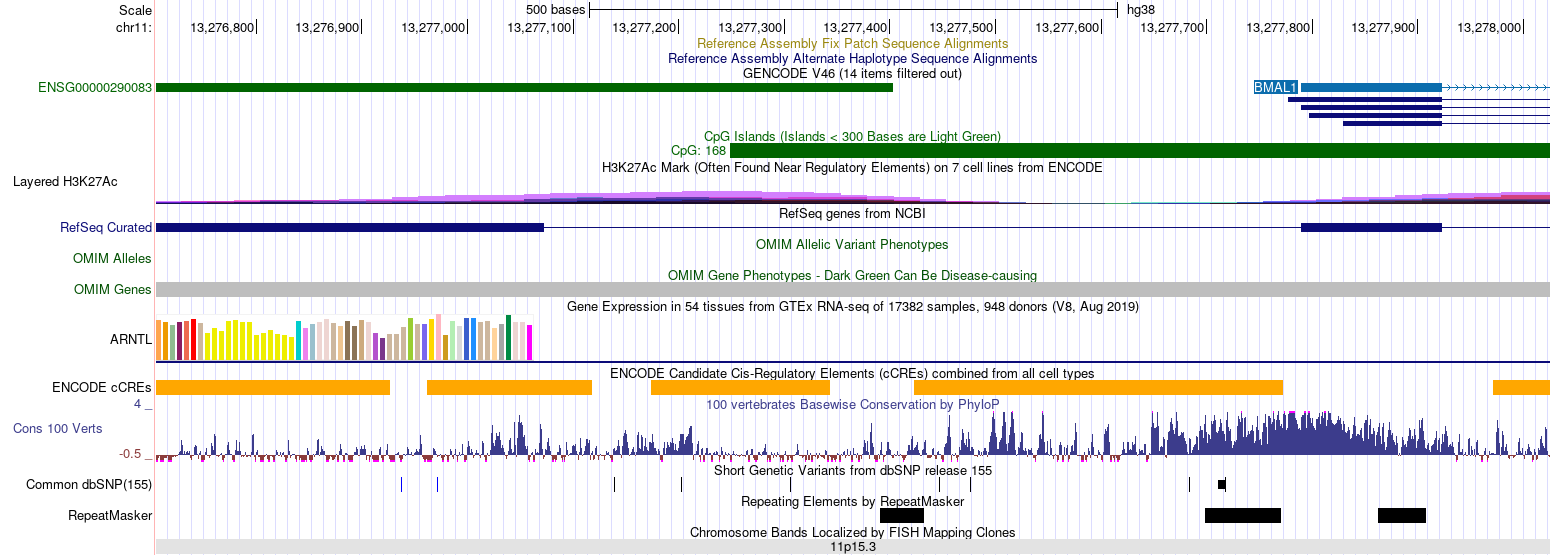


***BMAL1***


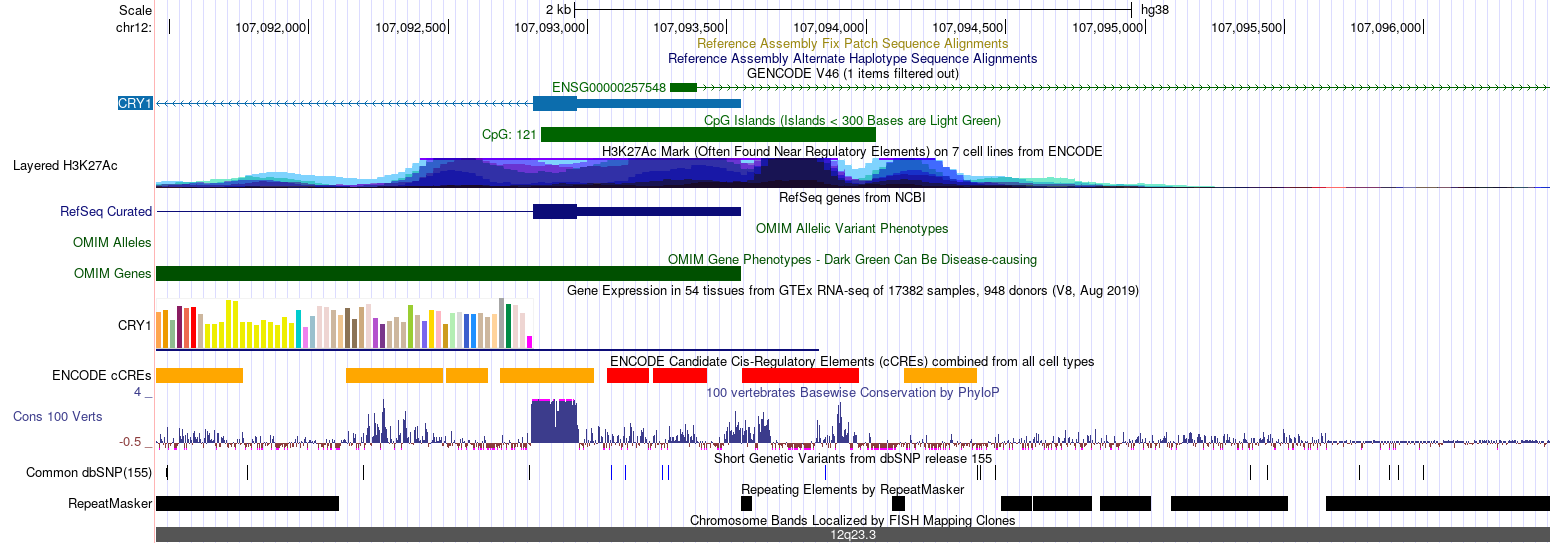


***CRY1***

**Figure S1.** *Continued.*


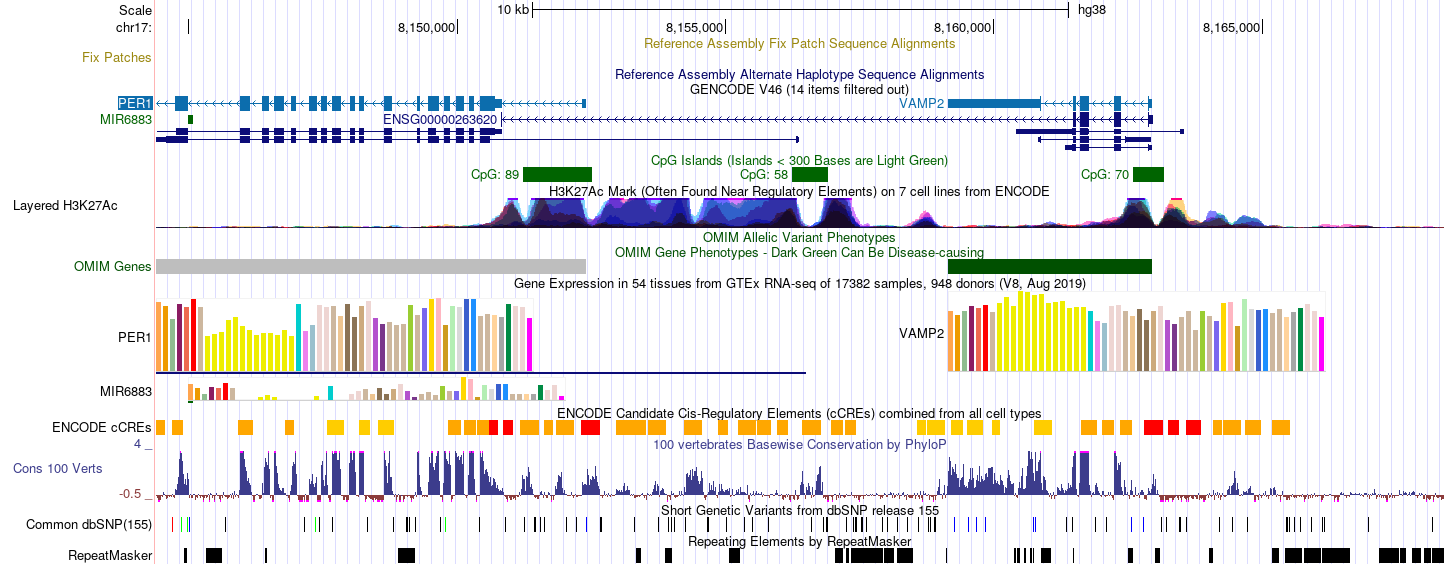


***PER1***


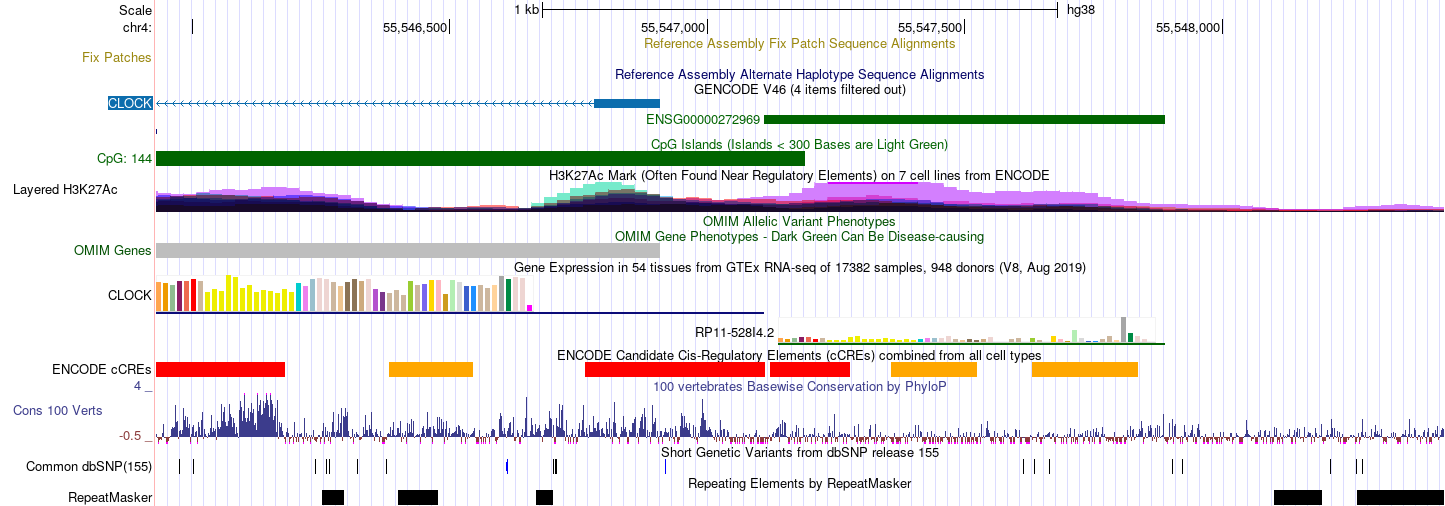


***CLOCK***


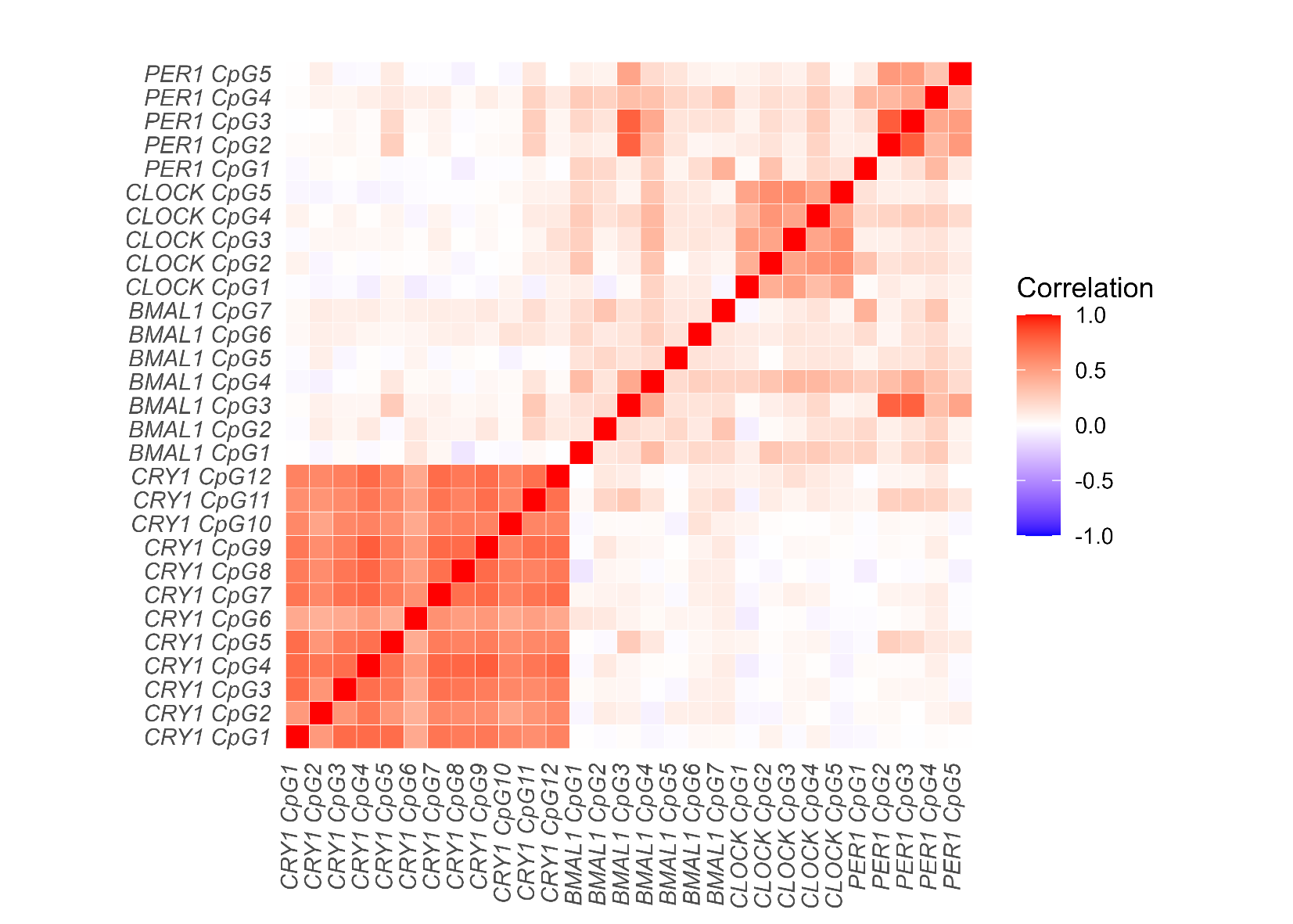
**Figure S2.** Spearman correlations between *CRY1*, *BMAL1*, *CLOCK*, and *PER1*.

| Table S2. Summary statistics of DNA methylation (%). | | | | | | | | | | | | | | | | |
| --- | --- | --- | --- | --- | --- | --- | --- | --- | --- | --- | --- | --- | --- | --- | --- | --- |
|  | **Total** | | | | | | **Non-cases** | | | | | **Cases** | | | | |
| Gene/CpG | **N** | **Mean (SD)** | **Min** | **Median** | **Max** | **N** | | **Mean (SD)** | **Min** | **Median** | **Max** | **N** | **Mean (SD)** | **Min** | **Median** | **Max** |
| *CRY1* CpG1 | 556 | 3.23 (3.55) | 0 | 3.05 | 55.10 | 270 | | 3.20 (2.84) | 0 | 3.01 | 16.87 | 286 | 3.27 (4.12) | 0 | 3.25 | 55.10 |
| CpG2 | 556 | 3.35 (3.77) | 0 | 3.03 | 53.00 | 270 | | 3.10 (2.94) | 0 | 2.77 | 16.65 | 286 | 3.59 (4.41) | 0 | 3.19 | 53.00 |
| CpG3 | 556 | 3.59 (3.78) | 0 | 3.36 | 55.48 | 270 | | 3.46 (3.09) | 0 | 3.30 | 16.92 | 286 | 3.71 (4.34) | 0 | 3.48 | 55.48 |
| CpG4 | 554 | 2.98 (3.63) | 0 | 2.77 | 53.57 | 270 | | 2.80 (2.96) | 0 | 2.53 | 15.99 | 284 | 3.15 (4.17) | 0 | 2.96 | 53.57 |
| CpG5 | 554 | 3.08 (3.78) | 0 | 2.83 | 56.21 | 270 | | 2.69 (2.89) | 0 | 2.48 | 13.96 | 284 | 3.45 (4.43) | 0 | 3.23 | 56.21 |
| CpG6 | 553 | 7.14 (5.24) | 0 | 6.58 | 71.33 | 269 | | 6.98 (5.37) | 0 | 6.31 | 71.33 | 284 | 7.29 (5.12) | 0 | 6.73 | 62.28 |
| CpG7 | 553 | 3.18 (3.39) | 0 | 2.92 | 51.55 | 269 | | 3.15 (2.70) | 0 | 2.85 | 13.28 | 284 | 3.22 (3.94) | 0 | 3.03 | 51.55 |
| CpG8 | 553 | 2.86 (3.17) | 0 | 2.64 | 27.89 | 269 | | 2.68 (2.93) | 0 | 2.33 | 14.61 | 284 | 3.03 (3.38) | 0 | 2.89 | 27.89 |
| CpG9 | 553 | 2.30 (4.14) | 0 | 0 | 68.62 | 269 | | 2.11 (2.89) | 0 | 0 | 13.16 | 284 | 2.48 (5.04) | 0 | 0 | 68.62 |
| CpG10 | 552 | 3.24 (3.97) | 0 | 3.13 | 54.81 | 268 | | 3.07 (2.84) | 0 | 3.08 | 14.84 | 284 | 3.39 (4.79) | 0 | 3.19 | 54.81 |
| CpG11 | 549 | 5.91 (4.97) | 0 | 5.91 | 74.92 | 267 | | 5.85 (3.79) | 0 | 5.98 | 18.21 | 282 | 5.97 (5.88) | 0 | 5.87 | 74.92 |
| CpG12 | 552 | 3.36 (3.18) | 0 | 3.37 | 47.26 | 268 | | 3.32 (2.67) | 0 | 3.36 | 14.31 | 284 | 3.40 (3.60) | 0 | 3.39 | 47.26 |
| *BMAL1* CpG1 | 589 | 1.99 (3.96) | 0 | 1.80 | 92.19 | 285 | | 1.76 (1.26) | 0 | 1.77 | 8.66 | 304 | 2.22 (5.38) | 0 | 1.85 | 92.19 |
| CpG2 | 589 | 1.03 (1.35) | 0 | 0 | 9.07 | 285 | | 1.14 (1.42) | 0 | 0 | 9.07 | 304 | 0.93 (1.28) | 0 | 0 | 8.63 |
| CpG3 | 589 | 3.71 (5.70) | 0 | 3.28 | 100 | 285 | | 3.25 (2.14) | 0 | 3.26 | 10.81 | 304 | 4.14 (7.64) | 0 | 3.33 | 100 |
| CpG4 | 588 | 1.20 (1.57) | 0 | 0 | 8.75 | 285 | | 1.16 (1.54) | 0 | 0 | 8.75 | 303 | 1.25 (1.60) | 0 | 0 | 8.36 |
| CpG5 | 588 | 0.57 (1.31) | 0 | 0 | 12.16 | 285 | | 0.60 (1.32) | 0 | 0 | 7.62 | 303 | 0.55 (1.31) | 0 | 0 | 12.16 |
| CpG6 | 558 | 5.00 (4.82) | 0 | 4.42 | 70.61 | 285 | | 5.05 (6.12) | 0 | 4.36 | 70.61 | 303 | 4.96 (3.14) | 0 | 4.50 | 28.69 |
| CpG7 | 589 | 3.60 (3.51) | 0 | 3.57 | 45.94 | 285 | | 3.81 (4.19) | 0 | 3.47 | 45.94 | 304 | 3.41 (2.70) | 0 | 3.58 | 21.77 |
| *CLOCK* CpG1 | 584 | 2.57 (3.39) | 0 | 2.91 | 46.64 | 283 | | 2.35 (3.54) | 0 | 2.73 | 46.64 | 301 | 2.78 (3.23) | 0 | 3.05 | 29.58 |
| CpG2 | 584 | 1.87 (2.71) | 0 | 0 | 25.56 | 283 | | 1.93 (2.85) | 0 | 0 | 25.56 | 301 | 1.82 (2.58) | 0 | 0 | 24.93 |
| CpG3 | 584 | 3.49 (3.64) | 0 | 3.60 | 42.58 | 283 | | 3.51 (3.84) | 0 | 3.58 | 42.58 | 301 | 3.47 (3.45) | 0 | 3.69 | 41.86 |
| CpG4 | 584 | 2.87 (3.53) | 0 | 3.12 | 48.34 | 283 | | 2.93 (4.07) | 0 | 3.04 | 48.34 | 301 | 2.81 (2.93) | 0 | 3.20 | 26.19 |
| CpG5 | 584 | 3.19 (3.30) | 0 | 3.51 | 44.01 | 283 | | 3.26 (3.65) | 0 | 3.48 | 44.01 | 301 | 3.12 (2.94) | 0 | 3.57 | 32.60 |
| *PER1* CpG1 | 555 | 0.88 (2.20) | 0 | 0 | 22.76 | 266 | | 0.91 (2.48) | 0 | 0 | 22.76 | 289 | 0.85 (1.92) | 0 | 0 | 16.42 |
| CpG2 | 555 | 1.58 (2.94) | 0 | 0 | 48.92 | 266 | | 1.48 (1.99) | 0 | 0 | 9.57 | 289 | 1.67 (3.61) | 0 | 0 | 48.92 |
| CpG3 | 554 | 1.45 (2.96) | 0 | 0 | 44.49 | 265 | | 1.25 (2.12) | 0 | 0 | 8.93 | 289 | 1.64 (3.55) | 0 | 0 | 44.49 |
| CpG4 | 554 | 1.70 (2.89) | 0 | 0 | 39.54 | 265 | | 1.53 (2.28) | 0 | 0 | 8.27 | 289 | 1.86 (3.35) | 0 | 0 | 39.54 |
| CpG5 | 554 | 0.15 (0.71) | 0 | 0 | 8.01 | 265 | | 0.12 (0.56) | 0 | 0 | 4.59 | 289 | 0.18 (0.82) | 0 | 0 | 8.01 |
| SD – standard deviation.  Min – minimum  Max - maximum | | | | | | | | | | | | | | | | |

| Table S3. Associations between 5-year mean road traffic noise at baseline (per 10 dB) and *CRY1*, *BMAL1*, *CLOCK*, and *PER1* methylation (M−values) in unadjusted linear regression models. | | | |
| --- | --- | --- | --- |
| Gene/CpG | **Crude Models^a^** | |  |
|  | **N** | **Beta (SE), *p-value*** |  |
| *CRY1* CpG1 | 556 | -0.11 (0.07), 0.11 |  |
| *CRY1* CpG2 | 556 | -0.13 (0.07), 0.04 |  |
| *CRY1* CpG3 | 556 | -0.06 (0.07), 0.35 |  |
| *CRY1* CpG4 | 554 | -0.08 (0.07), 0.22 |  |
| *CRY1* CpG5 | 554 | -0.02 (0.07), 0.77 |  |
| *CRY1* CpG6 | 553 | -0.06 (0.05), 0.25 |  |
| *CRY1* CpG7 | 553 | -0.01 (0.06), 0.88 |  |
| *CRY1* CpG8 | 553 | -0.04 (0.06), 0.57 |  |
| *CRY1* CpG9 | 553 | -0.10 (0.07), 0.18 |  |
| *CRY1* CpG10 | 552 | -0.07 (0.08), 0.41 |  |
| *CRY1* CpG11 | 549 | 0.01 (0.06), 0.99 |  |
| *CRY1* CpG12 | 552 | -0.08 (0.07), 0.23 |  |
| *BMAL1* CpG1 | 589 | -0.03 (0.06), 0.54 |  |
| *BMAL1* CpG2 | 589 | -0.11 (0.05), 0.03 |  |
| *BMAL1* CpG3 | 588 | -0.07 (0.06), 0.19 |  |
| *BMAL1* CpG4 | 588 | 0.02 (0.5), 0.71 |  |
| *BMAL1* CpG5 | 588 | -0.04 (0.05), 0.42 |  |
| *BMAL1* CpG6 | 588 | -0.14 (0.05), 0.005 |  |
| *BMAL1* CpG7 | 589 | -0.12 (0.06), 0.04 |  |
| *CLOCK* CpG1 | 584 | -0.02 (0.05), 0.69 |  |
| *CLOCK* CpG2 | 584 | -0.01 (0.06), 0.88 |  |
| *CLOCK* CpG3 | 584 | -0.03 (0.06), 0.59 |  |
| *CLOCK* CpG4 | 584 | -0.04 (0.06), 0.53 |  |
| *CLOCK* CpG5 | 584 | -0.05 (0.05), 0.36 |  |
| *PER1* CpG1 | 555 | -0.04 (0.05), 0.41 |  |
| *PER1* CpG2 | 555 | -0.04 (0.06), 0.54 |  |
| *PER1* CpG3 | 554 | 0.01 (0.06), 0.84 |  |
| *PER1* CpG4 | 554 | -0.06 (0.06), 0.36 |  |
| *PER1* CpG5 | 554 | -0.01 (0.02), 0.53 |  |
| ^a^ Adjusted for age.  SE – standard error. | | | |

| Table S4. Associations between *CRY1*, *BMAL1*, *CLOCK*, and *PER1* methylation (M−values) and breast cancer in unadjusted logistic regression models. | | |
| --- | --- | --- |
| Gene/CpG | **Continuous^a^** | |
|  | **N** | **OR (95% CI)** |
| *CRY1* CpG1 | 556 | 1.01 (0.89-1.15) |
| *CRY1* CpG2 | 556 | 0.90 (0.79-1.03) |
| *CRY1* CpG3 | 556 | 0.95 (0.84-1.09) |
| *CRY1* CpG4 | 554 | 0.92 (0.80-1.05) |
| *CRY1* CpG5 | 554 | 0.85 (0.75-0.96) |
| *CRY1* CpG6 | 553 | 0.89 (0.74-1.07) |
| *CRY1* CpG7 | 553 | 1.01 (0.87-1.17) |
| *CRY1* CpG8 | 553 | 0.90 (0.79-1.04) |
| *CRY1* CpG9 | 553 | 0.96 (0.84-1.09) |
| *CRY1* CpG10 | 552 | 0.98 (0.88-1.10) |
| *CRY1* CpG11 | 549 | 1.03 (0.88-1.20) |
| *CRY1* CpG12 | 552 | 0.99 (0.87-1.13) |
| *BMAL1* CpG1 | 589 | 0.88 (0.75-1.02) |
| *BMAL1* CpG2 | 589 | 1.19 (1.00-1.41) |
| *BMAL1* CpG3 | 588 | 0.91 (0.78-1.06) |
| *BMAL1* CpG4 | 588 | 0.94 (0.79-1.12) |
| *BMAL1* CpG5 | 588 | 1.04 (0.87-1.25) |
| *BMAL1* CpG6 | 588 | 0.92 (0.78-1.09) |
| *BMAL1* CpG7 | 589 | 1.07 (0.93-1.24) |
| *CLOCK* CpG1 | 584 | 0.83 (0.71-0.98) |
| *CLOCK* CpG2 | 584 | 1.02 (0.88-1.18) |
| *CLOCK* CpG3 | 584 | 0.98 (0.84-1.14) |
| *CLOCK* CpG4 | 584 | 0.99 (0.86-1.14) |
| *CLOCK* CpG5 | 584 | 1.01 (0.86-1.18) |
| *PER1* CpG1 | 555 | 1.00 (0.84-1.18) |
| *PER1* CpG2 | 555 | 1.00 (0.85-1.16) |
| *PER1* CpG3 | 554 | 0.91 (0.77-1.07) |
| *PER1* CpG4 | 554 | 0.93 (0.81-1.07) |
| *PER1* CpG5 | 554 | 0.85 (0.54-1.33) |
| ^a^ Adjusted for age.  OR – odds ratio.  CI – confidence interval. | | |

| Table S5. Associations between 5-year mean road traffic noise at baseline (per 10 dB) and *CRY1*, *BMAL1*, *CLOCK*, and *PER1* methylation (M−values) in adjusted linear regression models and the associations between gene methylation and breast cancer in adjusted logistic regression models additionally adjusted for PM_2.5_. | | | | |
| --- | --- | --- | --- | --- |
|  | **Road traffic noise and methylation** | | | **DNA methylation and breast cancer** |
| Gene/CpG | **All^a^** | **Non-cases^a^**  **n=292** | **Cases^a^**  **n=318** | **All^a^** |
|  | **Beta (SE), *p-value*** | **Beta (SE), *p-value*** | **Beta (SE), *p-value*** | **OR (95% CI)** |
| *CRY1* CpG1 | -0.15 (0.07), 0.03 | -0.05 (0.10), 0.58 | -0.25 (0.10), 0.02 | 0.98 (0.85-1.12) |
| *CRY1* CpG2 | -0.17 (0.07), 0.01 | -0.04 (0.10), 0.69 | -0.33 (0.10), <0.01 | 0.88 (0.76-1.02) |
| *CRY1* CpG3 | -0.07 (0.07), 0.35 | 0.04 (0.10), 0.66 | -0.19 (0.11), 0.08 | 0.94 (0.83-1.08) |
| *CRY1* CpG4 | -0.09 (0.07), 0.18 | 0.01 (0.10), 0.90 | -0.23 (0.10), 0.02 | 0.90 (0.79-1.04) |
| *CRY1* CpG5 | -0.03 (0.08), 0.72 | 0.01 (0.11), 0.97 | -0.06 (0.11), 0.63 | 0.84 (0.74-0.96) |
| *CRY1* CpG6 | -0.09 (0.05), 0.08 | 0.01 (0.08), 0.90 | -0.20 (0.07), <0.01 | 0.86 (0.71-1.04) |
| *CRY1* CpG7 | -0.03 (0.07), 0.61 | 0.07 (0.09), 0.47 | -0.15 (0.10), 0.11 | 1.02 (0.87-1.18) |
| *CRY1* CpG8 | -0.08 (0.07), 0.22 | 0.01 (0.10), 0.94 | -0.17 (0.10), 0.08 | 0.90 (0.78-1.04) |
| *CRY1* CpG9 | -0.12 (0.08), 0.13 | -0.04 (0.10), 0.72 | -0.22 (0.11), 0.06 | 0.94 (0.82-1.07) |
| *CRY1* CpG10 | -0.11 (0.08), 0.21 | -0.02 (0.12), 0.88 | -0.22 (0.13), 0.09 | 0.98 (0.87-1.10) |
| *CRY1* CpG11 | -0.05 (0.06), 0.42 | 0.05 (0.08), 0.52 | -0.16 (0.09), 0.09 | 1.02 (0.86-1.20) |
| *CRY1* CpG12 | -0.15 (0.07), 0.04 | -0.13 (0.10), 0.19 | -0.21 (0.10), 0.05 | 0.98 (0.85-1.13) |
| *BMAL1* CpG1 | -0.03 (0.06), 0.62 | 0.01 (0.08), 0.91 | -0.07 (0.09), 0.42 | 0.87 (0.75-1.02) |
| *BMAL1* CpG2 | -0.14 (0.05), 0.01 | -0.14 (0.08), 0.07 | -0.13 (0.07), 0.08 | 1.23 (1.03-1.47) |
| *BMAL1* CpG3 | -0.09 (0.06), 0.13 | -0.14 (0.08), 0.08 | -0.06 (0.09), 0.53 | 0.93 (0.79-1.08) |
| *BMAL1* CpG4 | 0.01 (0.05), 0.89 | -0.01 (0.07), 0.96 | 0.02 (0.07), 0.75 | 0.95 (0.79-1.14) |
| *BMAL1* CpG5 | -0.06 (0.05), 0.23 | -0.07 (0.07), 0.37 | -0.04 (0.07), 0.59 | 1.02 (0.84-1.22) |
| *BMAL1* CpG6 | -0.14 (0.05), 0.01 | -0.16 (0.08), 0.05 | -0.11 (0.07), 0.13 | 0.93 (0.79-1.11) |
| *BMAL1* CpG7 | -0.14 (0.06), 0.03 | -0.22 (0.09), 0.01 | -0.05 (0.09), 0.57 | 1.08 (0.93-1.25) |
| *CLOCK* CpG1 | -0.05 (0.06), 0.39 | -0.12 (0.08), 0.14 | 0.02 (0.08), 0.81 | 0.80 (0.68-0.94) |
| *CLOCK* CpG2 | -0.03 (0.06), 0.62 | 0.02 (0.09), 0.78 | -0.10 (0.08), 0.22 | 0.99 (0.85-1.16) |
| *CLOCK* CpG3 | -0.05 (0.06), 0.41 | -0.10 (0.09), 0.23 | 0.01 (0.08), 0.87 | 0.97 (0.84-1.14) |
| *CLOCK* CpG4 | -0.05 (0.06); 0.43 | -0.05 (0.09), 0.62 | -0.07 (0.09), 0.41 | 1.00 (0.86-1.16) |
| *CLOCK* CpG5 | -0.07 (0.06), 0.18 | -0.06 (0.08), 0.48 | -0.08 (0.08), 0.32 | 0.98 (0.83-1.16) |
| *PER1* CpG1 | -0.03 (0.06), 0.57 | -0.02 (0.08), 0.82 | -0.06 (0.08), 0.42 | 1.02 (0.86-1.21) |
| *PER1* CpG2 | -0.02 (0.06), 0.75 | 0.01 (0.09), 0.98 | -0.03 (0.09), 0.72 | 0.99 (0.85-1.16) |
| *PER1* CpG3 | 0.02 (0.06), 0.71 | 0.09 (0.08), 0.25 | -0.05 (0.09), 0.54 | 0.91 (0.77-1.07) |
| *PER1* CpG4 | -0.04 (0.07), 0.52 | 0.01 (0.09), 0.87 | -0.12 (0.10), 0.21 | 0.93 (0.80-1.08) |
| *PER1* CpG5 | -0.01 (0.02), 0.63 | -0.01 (0.03), 0.70 | -0.01 (0.03), 0.87 | 0.79 (0.49-1.27) |
| ^a^ Adjusted for age, parity, physical activity, education level, civil status, occupational status, smoking status, alcohol consumption, PM_2.5_.  OR – odds ratio.  CI – confidence interval.  SE – standard error. | | | | |

| Table S6. Associations between 5-year mean road traffic noise at baseline (per 10 dB) and *CRY1*, *BMAL1*, *CLOCK*, and *PER1* methylation (M−values) in adjusted linear regression models and the associations between gene methylation and breast cancer in adjusted logistic regression models additionally adjusted for NO_x_. | | | | |
| --- | --- | --- | --- | --- |
|  | **Road traffic noise and methylation** | | | **DNA methylation and breast cancer** |
| Gene/CpG | **All^a^** | **Non-cases^a^**  **n=292** | **Cases^a^**  **n=318** | **All^a^** |
|  | **Beta (SE), *p-value*** | **Beta (SE), *p-value*** | **Beta (SE), *p-value*** | **OR (95% CI)** |
| *CRY1* CpG1 | -0.20 (0.09), 0.02 | -0.10 (0.12), 0.41 | -0.29 (0.12), 0.02 | 0.98 (0.86-1.13) |
| *CRY1* CpG2 | -0.19 (0.08), 0.02 | -0.02 (0.12), 0.86 | -0.37 (0.12), <0.01 | 0.88 (0.77-1.02) |
| *CRY1* CpG3 | -0.08 (0.09), 0.38 | 0.10 (0.12), 0.44 | -0.24 (0.13), 0.06 | 0.95 (0.83-1.08) |
| *CRY1* CpG4 | -0.10 (0.08), 0.24 | 0.01 (0.12), 0.99 | -0.24 (0.12), 0.06 | 0.91 (0.79-1.04) |
| *CRY1* CpG5 | -0.06 (0.09), 0.50 | 0.03 (0.13), 0.80 | -0.15 (0.14), 0.26 | 0.84 (0.74-0.96) |
| *CRY1* CpG6 | -0.11 (0.06), 0.09 | -0.04 (0.09), 0.64 | -0.18 (0.09), 0.03 | 0.86 (0.71-1.05) |
| *CRY1* CpG7 | -0.02 (0.08), 0.80 | 0.09 (0.11), 0.43 | -0.15 (0.12), 0.21 | 1.02 (0.88-1.18) |
| *CRY1* CpG8 | -0.09 (0.08), 0.26 | 0.01 (0.12), 0.97 | -0.20 (0.12), 0.09 | 0.90 (0.78-1.04) |
| *CRY1* CpG9 | -0.07 (0.09), 0.42 | -0.02 (0.13), 0.86 | -0.15 (0.13), 0.27 | 0.94 (0.83-1.07) |
| *CRY1* CpG10 | -0.06 (0.10), 0.56 | 0.05 (0.14), 0.72 | -0.19 (0.15), 0.20 | 0.98 (0.87-1.10) |
| *CRY1* CpG11 | -0.02 (0.07), 0.74 | 0.06 (0.10), 0.52 | -0.12 (0.11), 0.27 | 1.03 (0.87-1.21) |
| *CRY1* CpG12 | -0.10 (0.09), 0.23 | -0.08 (0.12), 0.50 | -0.15 (0.12), 0.22 | 0.99 (0.86-1.13) |
| *BMAL1* CpG1 | -0.04 (0.07), 0.54 | -0.02 (0.10), 0.84 | -0.08 (0.11), 0.45 | 0.87 (0.75-1.02) |
| *BMAL1* CpG2 | -0.17 (0.06), <0.01 | -0.21 (0.09), 0.03 | -0.14 (0.09), 0.11 | 1.23 (1.03-1.47) |
| *BMAL1* CpG3 | -0.10 (0.07), 0.18 | -0.14 (0.10), 0.15 | -0.07 (0.11), 0.52 | 0.93 (0.79-1.09) |
| *BMAL1* CpG4 | 0.03 (0.06), 0.62 | 0.05 (0.09), 0.56 | 0.02 (0.09), 0.82 | 0.95 (0.79-1.14) |
| *BMAL1* CpG5 | -0.09 (0.06), 0.16 | -0.18 (0.09), 0.04 | 0.01 (0.08), 0.92 | 1.02 (0.84-1.23) |
| *BMAL1* CpG6 | -0.13 (0.07), 0.04 | -0.15 (0.10), 0.15 | -0.12 (0.09), 0.18 | 0.93 (0.79-1.11) |
| *BMAL1* CpG7 | -0.18 (0.07), 0.02 | -0.25 (0.11), 0.02 | -0.10 (0.10), 0.32 | 1.08 (0.93-1.25) |
| *CLOCK* CpG1 | -0.08 (0.07), 0.24 | -0.11 (0.10), 0.25 | -0.04 (0.10), 0.69 | 0.80 (0.68-0.94) |
| *CLOCK* CpG2 | 0.01 (0.07), 0.98 | -0.05 (0.11), 0.62 | 0.04 (0.10), 0.71 | 0.99 (0.86-1.16) |
| *CLOCK* CpG3 | -0.05 (0.07), 0.48 | -0.16 (0.11), 0.15 | 0.04 (0.10), 0.66 | 0.98 (0.84-1.14) |
| *CLOCK* CpG4 | -0.05 (0.07), 0.49 | -0.12 (0.11), 0.29 | -0.01 (0.10), 0.94 | 1.00 (0.87-1.16) |
| *CLOCK* CpG5 | -0.08 (0.07), 0.25 | -0.14 (0.10), 0.17 | -0.02 (0.09), 0.86 | 0.98 (0.83-1.16) |
| *PER1* CpG1 | 0.01 (0.07), 0.88 | 0.04 (0.10), 0.67 | -0.02 (0.10), 0.85 | 1.02 (0.86-1.21) |
| *PER1* CpG2 | 0.01 (0.07), 0.96 | 0.02 (0.10), 0.82 | -0.01 (0.11), 0.99 | 0.99 (0.85-1.16) |
| *PER1* CpG3 | 0.03 (0.07), 0.70 | 0.10 (0.10), 0.30 | -0.05 (0.11), 0.64 | 0.91 (0.77-1.07) |
| *PER1* CpG4 | -0.05 (0.08), 0.56 | 0.01 (0.11), 0.97 | -0.11 (0.12), 0.33 | 0.93 (0.80-1.08) |
| *PER1* CpG5 | -0.02 (0.03), 0.45 | -0.06 (0.03), 0.07 | 0.02 (0.04), 0.68 | 0.79 (0.49-1.26) |
| ^a^ Adjusted for age, parity, physical activity, education level, civil status, occupational status, smoking status, alcohol consumption, PM_2.5_.  OR – odds ratio.  CI – confidence interval.  SE – standard error. | | | | |

| Table S7. Associations between 5-year mean road traffic noise at baseline (per 10 dB) and *CRY1*, *BMAL1*, *CLOCK*, and *PER1* methylation (M−values) in adjusted linear regression models and the associations between gene methylation and breast cancer in adjusted logistic regression models additionally adjusted for inconvenient working hours. | | | | |
| --- | --- | --- | --- | --- |
|  | **Road traffic noise and methylation** | | | **DNA methylation and breast cancer** |
| Gene/CpG | **All^a^** | **Non-cases^a^**  **n=292** | **Cases^a^**  **n=318** | **All^a^** |
|  | **Beta (SE), *p-value*** | **Beta (SE), *p-value*** | **Beta (SE), *p-value*** | **OR (95% CI)** |
| *CRY1* CpG1 | -0.14 (0.07), 0.05 | -0.06 (0.10), 0.54 | -0.22 (0.10), 0.03 | 0.98 (0.85-1.12) |
| *CRY1* CpG2 | -0.17 (0.07), 0.01 | -0.06 (0.09), 0.56 | -0.31 (0.10), <0.01 | 0.87 (0.76-1.01) |
| *CRY1* CpG3 | -0.06 (0.07), 0.44 | 0.05 (0.10), 0.59 | -0.18 (0.11), 0.09 | 0.95 (0.83-1.09) |
| *CRY1* CpG4 | -0.08 (0.07), 0.24 | 0.01 (0.10), 0.90 | -0.22 (0.10), 0.03 | 0.90 (0.78-1.04) |
| *CRY1* CpG5 | -0.04 (0.08), 0.62 | 0.01 (0.11), 0.99 | -0.08 (0.11), 0.51 | 0.84 (0.74-0.95) |
| *CRY1* CpG6 | -0.09 (0.05), 0.08 | -0.01 (0.08), 0.98 | -0.19 (0.07), <0.01 | 0.85 (0.70-1.03) |
| *CRY1* CpG7 | -0.03 (0.06), 0.68 | 0.07 (0.09), 0.47 | -0.14 (0.10), 0.14 | 1.01 (0.87-1.18) |
| *CRY1* CpG8 | -0.08 (0.07), 0.25 | 0.01 (0.10), 0.89 | -0.17 (0.10), 0.08 | 0.90 (0.77-1.04) |
| *CRY1* CpG9 | -0.12 (0.07), 0.11 | -0.04 (0.10), 0.70 | -0.22 (0.11), 0.05 | 0.93 (0.82-1.06) |
| *CRY1* CpG10 | -0.12 (0.08), 0.17 | -0.02 (0.12), 0.84 | -0.23 (0.13), 0.06 | 0.97 (0.86-1.09) |
| *CRY1* CpG11 | -0.04 (0.06), 0.48 | 0.07 (0.08), 0.41 | -0.16 (0.09), 0.07 | 1.01 (0.86-1.19) |
| *CRY1* CpG12 | -0.14 (0.07), 0.04 | -0.12 (0.10), 0.22 | -0.22 (0.10), 0.04 | 0.97 (0.84-1.11) |
| *BMAL1* CpG1 | -0.03 (0.06), 0.62 | 0.04 (0.08), 0.63 | -0.11 (0.09), 0.22 | 0.87 (0.74-1.02) |
| *BMAL1* CpG2 | -0.13 (0.05), 0.01 | -0.14 (0.08), 0.07 | -0.13 (0.07), 0.08 | 1.22 (1.02-1.46) |
| *BMAL1* CpG3 | -0.08 (0.06), 0.15 | -0.13 (0.08), 0.10 | -0.06 (0.09), 0.46 | 0.91 (0.78-1.07) |
| *BMAL1* CpG4 | -0.01 (0.05), 0.87 | -0.01 (0.07), 0.89 | 0.01 (0.07), 0.99 | 0.92 (0.77-1.11) |
| *BMAL1* CpG5 | -0.05 (0.05), 0.35 | -0.05 (0.07), 0.49 | -0.02 (0.07), 0.74 | 1.00 (0.83-1.21) |
| *BMAL1* CpG6 | -0.14 (0.05), 0.01 | -0.15 (0.08), 0.06 | -0.13 (0.07), 0.07 | 0.92 (0.78-1.10) |
| *BMAL1* CpG7 | -0.13 (0.06), 0.03 | -0.20 (0.09), 0.02 | -0.07 (0.08), 0.38 | 1.07 (0.92-1.24) |
| *CLOCK* CpG1 | -0.04 (0.06), 0.47 | -0.12 (0.08), 0.15 | 0.03 (0.08), 0.74 | 0.79 (0.67-0.94) |
| *CLOCK* CpG2 | -0.03 (0.06), 0.63 | 0.04 (0.09), 0.64 | -0.13 (0.08), 0.12 | 0.99 (0.85-1.16) |
| *CLOCK* CpG3 | -0.05 (0.06), 0.44 | -0.08 (0.09), 0.33 | -0.01 (0.08), 0.94 | 0.97 (0.83-1.13) |
| *CLOCK* CpG4 | -0.04 (0.06); 0.54 | -0.01 (0.09), 0.89 | -0.08 (0.09), 0.35 | 1.00 (0.86-1.16) |
| *CLOCK* CpG5 | -0.08 (0.06), 0.16 | -0.05 (0.08), 0.53 | -0.10 (0.08), 0.20 | 0.97 (0.83-1.15) |
| *PER1* CpG1 | -0.03 (0.06), 0.57 | -0.01 (0.08), 0.89 | -0.06 (0.08), 0.40 | 1.01 (0.85-1.20) |
| *PER1* CpG2 | -0.01 (0.06), 0.81 | 0.01 (0.08), 0.88 | -0.03 (0.09), 0.74 | 0.98 (0.84-1.15) |
| *PER1* CpG3 | 0.02 (0.06), 0.73 | 0.11 (0.08), 0.17 | -0.08 (0.09), 0.40 | 0.91 (0.77-1.07) |
| *PER1* CpG4 | -0.06 (0.07), 0.40 | 0.03 (0.09), 0.76 | -0.15 (0.10), 0.12 | 0.93 (0.80-1.08) |
| *PER1* CpG5 | -0.02 (0.02), 0.48 | -0.01 (0.03), 0.64 | -0.01 (0.03), 0.73 | 0.77 (0.48-1.24) |
| ^a^ Adjusted for age, parity, physical activity, education level, civil status, occupational status, smoking status, alcohol consumption, inconvenient working hours.  OR – odds ratio.  CI – confidence interval.  SE – standard error. | | | | |

| Table S8. Associations between 5-year mean road traffic noise at baseline (per 10 dB) and *CRY1*, *BMAL1*, *CLOCK*, and *PER1* methylation (M−values) in adjusted linear regression models and the associations between gene methylation and breast cancer in adjusted logistic regression models additionally adjusted for BMI. | | | | |
| --- | --- | --- | --- | --- |
|  | **Road traffic noise and methylation** | | | **DNA methylation and breast cancer** |
| Gene/CpG | **All^a^** | **Non-cases^a^**  **n=292** | **Cases^a^**  **n=318** | **All^a^** |
|  | **Beta (SE), *p-value*** | **Beta (SE), *p-value*** | **Beta (SE), *p-value*** | **OR (95% CI)** |
| *CRY1* CpG1 | -0.13 (0.07), 0.06 | -0.05 (0.10), 0.60 | -0.22 (0.10), 0.03 | 0.98 (0.86-1.13) |
| *CRY1* CpG2 | -0.16 (0.07), 0.02 | -0.04 (0.10), 0.70 | -0.31 (0.10), <0.01 | 0.89 (0.76-1.02) |
| *CRY1* CpG3 | -0.06 (0.07), 0.43 | 0.05 (0.10), 0.63 | -0.17 (0.10), 0.10 | 0.95 (0.83-1.08) |
| *CRY1* CpG4 | -0.08 (0.07), 0.27 | 0.03 (0.10), 0.74 | -0.22 (0.10), 0.03 | 0.91 (0.79-1.05) |
| *CRY1* CpG5 | -0.03 (0.08), 0.73 | 0.01 (0.11), 0.92 | -0.05 (0.11), 0.65 | 0.84 (0.74-0.96) |
| *CRY1* CpG6 | -0.09 (0.05), 0.08 | 0.01 (0.08), 0.99 | -0.19 (0.07), <0.01 | 0.86 (0.71-1.04) |
| *CRY1* CpG7 | -0.02 (0.06), 0.70 | 0.07 (0.09), 0.45 | -0.14 (0.09), 0.14 | 1.02 (0.88-1.18) |
| *CRY1* CpG8 | -0.08 (0.07), 0.25 | 0.01 (0.10), 0.88 | -0.17 (0.10), 0.09 | 0.90 (0.78-1.04) |
| *CRY1* CpG9 | -0.12 (0.07), 0.12 | -0.03 (0.10), 0.79 | -0.21 (0.11), 0.05 | 0.94 (0.82-1.07) |
| *CRY1* CpG10 | -0.10 (0.08), 0.26 | -0.01 (0.12), 0.99 | -0.21 (0.12), 0.09 | 0.98 (0.87-1.10) |
| *CRY1* CpG11 | -0.04 (0.06), 0.53 | 0.07 (0.08), 0.35 | -0.16 (0.09), 0.09 | 1.03 (0.87-1.21) |
| *CRY1* CpG12 | -0.13 (0.07), 0.06 | -0.10 (0.10), 0.30 | -0.21 (0.10), 0.05 | 0.98 (0.86-1.13) |
| *BMAL1* CpG1 | -0.02 (0.06), 0.76 | 0.04 (0.08), 0.65 | -0.08 (0.09), 0.37 | 0.88 (0.75-1.03) |
| *BMAL1* CpG2 | -0.13 (0.05), 0.01 | -0.11 (0.08), 0.13 | -0.12 (0.07), 0.08 | 1.24 (1.03-1.48) |
| *BMAL1* CpG3 | -0.08 (0.06), 0.18 | -0.13 (0.08), 0.10 | -0.04 (0.09), 0.61 | 0.93 (0.79-1.09) |
| *BMAL1* CpG4 | 0.01 (0.05), 0.84 | -0.01 (0.07), 0.95 | 0.03 (0.07), 0.72 | 0.95 (0.79-1.14) |
| *BMAL1* CpG5 | -0.04 (0.05), 0.41 | -0.04 (0.07), 0.54 | -0.02 (0.07), 0.75 | 1.02 (0.85-1.23) |
| *BMAL1* CpG6 | -0.13 (0.05), 0.01 | -0.15 (0.08), 0.06 | -0.11 (0.07), 0.11 | 0.93 (0.78-1.10) |
| *BMAL1* CpG7 | -0.12 (0.06), 0.05 | -0.17 (0.09), 0.05 | -0.05 (0.08), 0.59 | 1.08 (0.93-1.26) |
| *CLOCK* CpG1 | -0.04 (0.06), 0.52 | -0.11 (0.08), 0.16 | 0.04 (0.08), 0.65 | 0.80 (0.68-0.94) |
| *CLOCK* CpG2 | -0.02 (0.06), 0.80 | 0.06 (0.09), 0.48 | -0.11 (0.08), 0.18 | 0.99 (0.86-1.16) |
| *CLOCK* CpG3 | -0.03 (0.06), 0.59 | -0.08 (0.09), 0.37 | 0.02 (0.08), 0.79 | 0.98 (0.84-1.14) |
| *CLOCK* CpG4 | -0.02 (0.06); 0.69 | 0.01 (0.09), 0.95 | -0.07 (0.09), 0.38 | 1.01 (0.87-1.17) |
| *CLOCK* CpG5 | -0.07 (0.06), 0.21 | -0.03 (0.08), 0.71 | -0.09 (0.08), 0.24 | 0.98 (0.84-1.16) |
| *PER1* CpG1 | -0.02 (0.06), 0.67 | 0.01 (0.08), 0.92 | -0.07 (0.08), 0.36 | 1.02 (0.86-1.22) |
| *PER1* CpG2 | -0.02 (0.06), 0.78 | 0.02 (0.08), 0.78 | -0.04 (0.09), 0.62 | 0.99 (0.85-1.16) |
| *PER1* CpG3 | 0.03 (0.06), 0.67 | 0.12 (0.08), 0.13 | -0.08 (0.09), 0.39 | 0.91 (0.77-1.07) |
| *PER1* CpG4 | -0.05 (0.07), 0.47 | 0.04 (0.09), 0.63 | -0.16 (0.10), 0.10 | 0.93 (0.80-1.08) |
| *PER1* CpG5 | -0.01 (0.02), 0.51 | -0.01 (0.03), 0.66 | -0.01 (0.03), 0.77 | 0.79 (0.49-1.26) |
| ^a^ Adjusted for age, parity, physical activity, education level, civil status, occupational status, smoking status, alcohol consumption, BMI.  OR – odds ratio.  CI – confidence interval.  SE – standard error. | | | | |

| Table S9. Associations between difficulty *falling* asleep and DNA methylation (M−values) of *CRY1*, *BMAL1*, *CLOCK*, and *PER1* in adjusted linear regression models (n=413). | | | | |
| --- | --- | --- | --- | --- |
|  | **Problems falling asleep at night^a^** | | | |
| Gene/CpG | **None**  **n=186** | **Small**  **n=104** | **Moderate**  **n=87** | **Large**  **n=36** |
|  | **Beta (SE), *p-value*** | **Beta (SE), *p-value*** | **Beta (SE), *p-value*** | **Beta (SE), *p-value*** |
| *CRY1* CpG1 | Reference | 0.30 (0.17), 0.08 | 0.01 (0.19), 0.94 | -0.07 (0.27), 0.79 |
| *CRY1* CpG2 | Reference | 0.04 (0.17), 0.83 | -0.25 (0.18), 0.17 | 0.03 (0.26), 0.91 |
| *CRY1* CpG3 | Reference | 0.51 (0.17), <0.01 | 0.16 (0.19), 0.39 | 0.11 (0.27), 0.68 |
| *CRY1* CpG4 | Reference | 0.42 (0.17), 0.01 | -0.10 (0.18), 0.59 | 0.12 (0.26), 0.64 |
| *CRY1* CpG5 | Reference | 0.38 (0.19), 0.04 | -0.18 (0.20), 0.39 | 0.07 (0.29), 0.81 |
| *CRY1* CpG6 | Reference | 0.17 (0.13), 0.18 | -0.30 (0.14), 0.03 | 0.03 (0.20), 0.89 |
| *CRY1* CpG7 | Reference | 0.38 (0.15), 0.01 | -0.08 (0.17), 0.64 | 0.10 (0.24), 0.66 |
| *CRY1* CpG8 | Reference | 0.45 (0.16), <0.01 | -0.15 (0.17), 0.38 | 0.39 (0.25), 0.11 |
| *CRY1* CpG9 | Reference | 0.13 (0.18), 0.46 | -0.33 (0.20), 0.09 | 0.13 (0.28), 0.64 |
| *CRY1* CpG10 | Reference | 0.45 (0.20), 0.02 | 0.04 (0.22), 0.87 | 0.33 (0.31), 0.29 |
| *CRY1* CpG11 | Reference | 0.12 (0.15), 0.40 | -0.01 (0.16), 0.98 | -0.09 (0.23), 0.68 |
| *CRY1* CpG12 | Reference | 0.07 (0.17), 0.67 | -0.17 (0.19), 0.36 | 0.10 (0.27), 0.71 |
| *BMAL1* CpG1 | Reference | 0.07 (0.13), 0.57 | -0.07 (0.14), 0.63 | -0.10 (0.20), 0.63 |
| *BMAL1* CpG2 | Reference | 0.07 (0.12), 0.57 | -0.05 (0.13), 0.73 | -0.02 (0.19), 0.91 |
| *BMAL1* CpG3 | Reference | 0.12 (0.14), 0.39 | 0.21 (0.15), 0.17 | 0.05 (0.22), 0.82 |
| *BMAL1* CpG4 | Reference | 0.02 (0.12), 0.85 | -0.01 (0.13), 0.95 | -0.22 (0.18), 0.23 |
| *BMAL1* CpG5 | Reference | -0.15 (0.12), 0.23 | -0.17 (0.14), 0.23 | 0.18 (0.20), 0.37 |
| *BMAL1* CpG6 | Reference | 0.10 (0.12), 0.40 | -0.05 (0.13), 0.70 | -0.07 (0.18), 0.72 |
| *BMAL1* CpG7 | Reference | -0.07 (0.14), 0.60 | -0.07 (0.15), 0.66 | -0.12 (0.21), 0.58 |
| *CLOCK* CpG1 | Reference | -0.11 (0.14), 0.43 | -0.07 (0.15), 0.66 | -0.01 (0.22), 0.99 |
| *CLOCK* CpG2 | Reference | -0.04 (0.14), 0.80 | 0.08 (0.16), 0.61 | -0.43 (0.23), 0.06 |
| *CLOCK* CpG3 | Reference | 0.12 (0.15), 0.42 | 0.17 (0.16), 0.29 | 0.10 (0.23), 0.68 |
| *CLOCK* CpG4 | Reference | 0.06 (0.15), 0.70 | -0.03 (0.17), 0.86 | 0.06 (0.24), 0.81 |
| *CLOCK* CpG5 | Reference | -0.24 (0.13), 0.08 | -0.27 (0.14), 0.06 | -0.32 (0.21), 0.13 |
| *PER1* CpG1 | Reference | 0.01 (0.14), 0.97 | 0.18 (0.16), 0.25 | -0.17 (0.22), 0.44 |
| *PER1* CpG2 | Reference | 0.11 (0.15), 0.48 | 0.04 (0.17), 0.78 | -0.07 (0.23), 0.78 |
| *PER1* CpG3 | Reference | 0.14 (0.15), 0.35 | 0.16 (0.16), 0.30 | 0.01 (0.22), 0.98 |
| *PER1* CpG4 | Reference | -0.11 (0.16), 0.49 | -0.24 (0.18), 0.17 | -0.04 (0.25), 0.87 |
| *PER1* CpG5 | Reference | -0.01 (0.06), 0.82 | 0.07 (0.07), 0.31 | 0.05 (0.09), 0.55 |
| ^a^ Adjusted for age, parity, physical activity, education level, civil status, occupational status, smoking status, and alcohol consumption.  OR – odds ratio.  CI – confidence interval.  SE – standard error. | | | | |

| Table S10. Associations between difficulty *staying* asleep and DNA methylation (M−values) of *CRY1*, *BMAL1*, *CLOCK*, and *PER1* in adjusted linear regression models (n=413). | | | | |
| --- | --- | --- | --- | --- |
|  | **Problems staying asleep at night^a^** | | | |
| Gene/CpG | **None**  **n=186** | **Small**  **n=104** | **Moderate**  **n=87** | **Large**  **n=36** |
|  | **Beta (SE), *p-value*** | **Beta (SE), *p-value*** | **Beta (SE), *p-value*** | **Beta (SE), *p-value*** |
| *CRY1* CpG1 | Reference | 0.15 (0.18), 0.39 | -0.01 (0.19), 0.96 | 0.50 (0.28), 0.07 |
| *CRY1* CpG2 | Reference | 0.19 (0.17), 0.27 | 0.01 (0.18), 0.94 | 0.31 (0.28), 0.27 |
| *CRY1* CpG3 | Reference | 0.03 (0.18), 0.89 | 0.02 (0.19), 0.93 | 0.30 (0.28), 0.29 |
| *CRY1* CpG4 | Reference | 0.27 (0.17), 0.12 | 0.01 (0.18), 0.97 | 0.51 (0.28), 0.07 |
| *CRY1* CpG5 | Reference | 0.24 (0.19), 0.21 | 0.06 (0.20), 0.76 | 0.77 (0.31), 0.01 |
| *CRY1* CpG6 | Reference | 0.21 (0.13), 0.13 | 0.14 (0.14), 0.33 | 0.29 (0.21), 0.17 |
| *CRY1* CpG7 | Reference | 0.06 (0.16), 0.72 | -0.05 (0.17), 0.79 | 0.01 (0.26), 0.98 |
| *CRY1* CpG8 | Reference | 0.19 (0.17), 0.26 | 0.18 (0.18), 0.30 | 0.51 (0.26), 0.05 |
| *CRY1* CpG9 | Reference | 0.22 (0.18), 0.24 | -0.07 (0.20), 0.24 | 0.31 (0.29), 0.29 |
| *CRY1* CpG10 | Reference | 0.31 (0.21), 0.14 | 0.30 (0.22), 0.17 | 0.66 (0.33), 0.05 |
| *CRY1* CpG11 | Reference | 0.19 (0.15), 0.22 | 0.16 (0.16), 0.33 | 0.09 (0.24), 0.70 |
| *CRY1* CpG12 | Reference | 0.01 (0.18), 0.96 | -0.11 (0.19), 0.56 | 0.06 (0.28), 0.84 |
| *BMAL1* CpG1 | Reference | -0.03 (0.13), 0.83 | 0.11 (0.14), 0.43 | -0.08 (0.21), 0.69 |
| *BMAL1* CpG2 | Reference | 0.19 (0.13), 0.14 | 0.26 (0.13), 0.05 | -0.34 (0.20), 0.09 |
| *BMAL1* CpG3 | Reference | 0.12 (0.15), 0.41 | 0.25 (0.15), 0.11 | 0.19 (0.23), 0.41 |
| *BMAL1* CpG4 | Reference | 0.16 (0.12), 0.20 | -0.05 (0.13), 0.68 | -0.02 (0.19), 0.91 |
| *BMAL1* CpG5 | Reference | 0.05 (0.13), 0.68 | -0.05 (0.14), 0.69 | -0.24 (0.21), 0.24 |
| *BMAL1* CpG6 | Reference | 0.21 (0.12), 0.08 | 0.20 (0.13), 0.11 | -0.16 (0.19), 0.42 |
| *BMAL1* CpG7 | Reference | 0.14 (0.14), 0.31 | 0.14 (0.15), 0.36 | -0.15 (0.22), 0.51 |
| *CLOCK* CpG1 | Reference | 0.04 (0.15), 0.77 | 0.05 (0.15), 0.73 | 0.15 (0.23), 0.53 |
| *CLOCK* CpG2 | Reference | 0.12 (0.15), 0.41 | 0.07 (0.16), 0.64 | -0.07 (0.24), 0.79 |
| *CLOCK* CpG3 | Reference | -0.01 (0.15), 0.96 | 0.16 (0.16), 0.33 | -0.21 (0.25), 0.40 |
| *CLOCK* CpG4 | Reference | -0.08 (0.16), 0.63 | -0.01 (0.17), 0.94 | -0.16 (0.26), 0.55 |
| *CLOCK* CpG5 | Reference | 0.02 (0.14), 0.86 | -0.13 (0.15), 0.38 | -0.13 (0.22), 0.56 |
| *PER1* CpG1 | Reference | 0.07 (0.15), 0.65 | 0.21 (0.16), 0.17 | -0.17 (0.23), 0.47 |
| *PER1* CpG2 | Reference | -0.07 (0.16), 0.68 | -0.11 (0.17), 0.50 | 0.26 (0.24), 0.29 |
| *PER1* CpG3 | Reference | 0.10 (0.15), 0.52 | 0.10 (0.16), 0.55 | 0.01 (0.23), 0.94 |
| *PER1* CpG4 | Reference | 0.14 (0.17), 0.41 | -0.01 (0.18), 0.93 | 0.09 (0.26), 0.72 |
| *PER1* CpG5 | Reference | 0.01 (0.06), 0.90 | 0.06 (0.07), 0.32 | -0.01 (0.10), 0.95 |
| ^a^ Adjusted for age, parity, physical activity, education level, civil status, occupational status, smoking status, and alcohol consumption.  OR – odds ratio.  CI – confidence interval.  SE – standard error. | | | | |

| Table S11. Association between trouble sleeping and risk of breast cancer (n=413). | |
| --- | --- |
| Difficulty falling asleep | **Adjusted Models^a^** |
|  | **OR (95% CI)** |
| None | Reference |
| Small | 1.16 (0.69-1.93) |
| Moderate | 1.22 (0.71-2.10) |
| Large | 2.07 (0.93-4.64) |
| Difficulty staying asleep |  |
| None | Reference |
| Small | 0.74 (0.43-1.27) |
| Moderate | 0.92 (0.53-1.61) |
| Large | 1.07 (0.46-2.50) |
| ^a^ Adjusted for age, parity, physical activity, education level, civil status, occupational status, smoking status, and alcohol consumption.  CI – confidence interval. | |
